# Supplementary material for: A Janus carbaporphyrin pseudo-dimer
Source: Nat Commun. 2024 Apr 4;15:2913. doi: 10.1038/s41467-024-47239-y (PMC10994945; doi:10.1038/s41467-024-47239-y)
Supplement: Supplementary file 1 — Supplementary Information [file 41467_2024_47239_MOESM1_ESM.pdf]

Supplementary Information

A Janus Carbaporphyrin Pseudo-dimer

Haodan He[1], Jiyeon Lee[2], Zhaohui Zong[1], Jiwon Kim[2], Vincent M. Lynch[3], Juwon Oh[4]*, Dongho Kim[5]*, Jonathan L. Sessler[3]* & Xian-Sheng Ke[1]*

Table of Contents

1. Synthesis and characterization
2. Photophysical measurements
3. Electrochemistry
4. Quantum chemical calculations
5. X-ray crystallography
6. ^{1}H, ^{13}C, ^{19}F, 2D-COSY NMR, HR-MALDI-TOF-MS and HR-ESI-MS spectra
7. Supplementary references

1. Synthesis and characterization

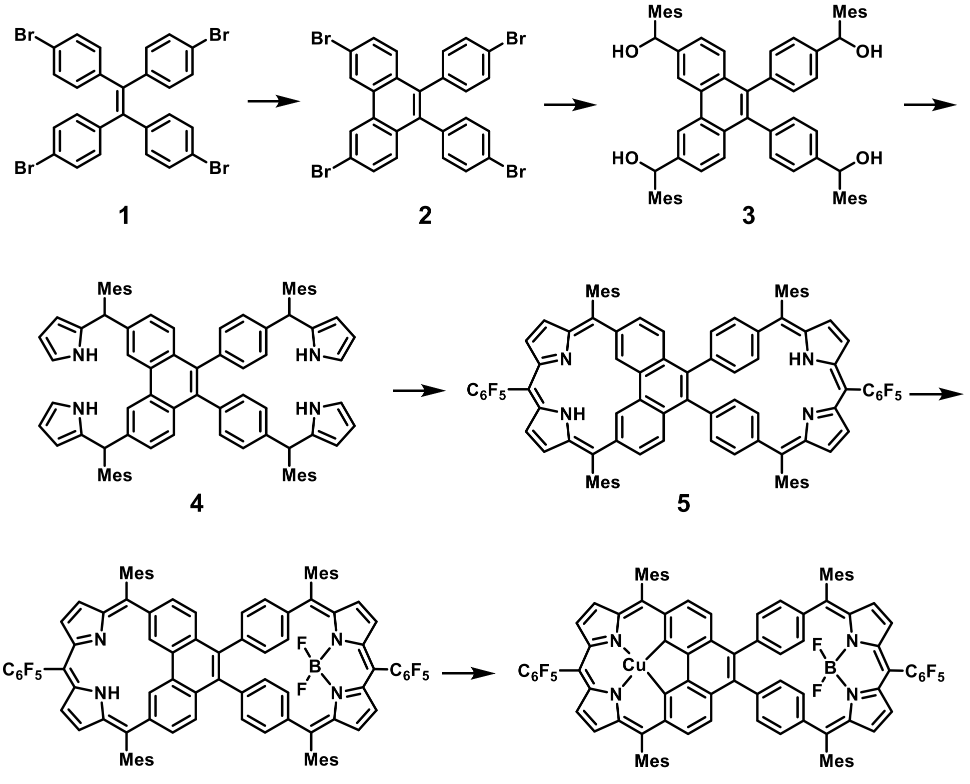

Supplementary Figure 1. Synthetic routes to **5** and **7**.

Synthesis of compound 2

Compound **1** (4.0 g, 6.2 mmol) and DDQ (1.4 g, 6.2 mmol) were dissolved in DCM (100 mL) in a nitrogen atmosphere at 0 °C, followed by the addition of trifluoromethanesulfonic acid (0.4 mL) for 0.5 h. The reaction temperature was then allowed to revert to room temperature and the reaction mixture stirred for another 12 h. After quenching the reaction with 5 mL methanol, 100 mL of a saturated potassium carbonate solution was added. The organic phase was separated and washed with water three times. Finally, the organic phase was concentrated under reduced pressure, and the product was recrystallized from n-hexane/DCM. Yield: 1.2 g (30%).^{1}H NMR (400 MHz, $CDCl_3$) δ 8.82 (s, 2H), 7.60 (d, J = 8.8 Hz, 2H), 7.41 (s, 4H), 7.35 (d, J = 8.8 Hz, 2H), 6.98 (d, J = 8.3 Hz, 4H); ^{13}C NMR (100 MHz, $CDCl_3$) δ 137.4, 136.2, 132.5, 131.5, 130.8, 130.6, 130.5, 129.5, 125.6, 121.9, 121.6; HRMS (APCI$^+$) *m/z* [M]$^+$: Calcd for $C_{26}H_{14}Br_4^+$ 641.7824, found: 641.7823.

Synthesis of compound 3

Compound **2** (3.3 g, 5.1 mmol) was dissolved in 200 mL dry THF under a nitrogen atmosphere, and stirred in a low-temperature reactor at -78 °C for 10 minutes. Then, 18.5 mL *n*-butyl lithium (2.5 M in hexanes) was slowly added. One hour later, 13.9 mL mesitylaldehyde were added. After one hour, the reaction was allowed to warm to room temperature, and 10 mL water were added to quench the reaction. The insoluble solids were removed by filtration. The filtrate was collected and the volatiles removed using a rotary evaporator. Further purification by column chromatography over silica gel (PE/EA: 4/1 as the eluent) gave 3.3 g of **3** as a white solid. Yield: 71%. ^{1}H NMR (600 MHz, $CDCl_3$) δ 8.90 – 8.89 (m, 1H), 8.79 (s, 1H), 7.46 (dd, J = 8.6, 3.8 Hz, 2H), 7.25 – 7.21 (m, 1H), 7.21 – 7.16 (m, 2H), 7.14 – 7.10 (m, 1H), 7.08 – 7.05

(m, 3H), 7.02 – 6.99 (m, 3H), 6.87 (s, 4H), 6.85 (d, *J* = 6.2 Hz, 4H), 6.53 (s, 2H), 6.27 (s, 2H), 2.31 (s, 6H), 2.29 – 2.27 (m, 12H), 2.26 (s, 6H), 2.19 (s, 6H), 2.17 (s, 6H); ^{13}C NMR (150 MHz, $CDCl_3$) *δ* 141.3, 141.2, 137.9, 137.9, 137.4, 137.4, 137.2, 137.1, 137.1, 131.0, 130.9, 130.9, 130.2, 130.1, 130.1, 127.8, 127.7, 124.7, 124.7, 124.6, 119.4, 119.3, 71.6, 71.5, 71.0, 21.0, 21.0, 20.8, 20.7, 20.7, 20.6; HRMS (ESI$^+$) *m/z* $[M+NH_4]^+$: Calcd for $C_{66}H_{70}NO_4^+$ 940.5299, found: 940.5305.

Synthesis of compound 4

In a 100 mL round-bottomed flask equipped with a reflux condenser and magnetic stirring, precursor **3** (1.5 g, 1.6 mmol) was added along with dry pyrrole (20 mL). After dissolution, the solution was purged with nitrogen for 20 minutes. Subsequently, boron trifluoride diethyl etherate (0.5 mL) was added and the solution was heated at reflux for 20 h under a nitrogen atmosphere. The solution was neutralized via the addition of triethylamine (1.5 mL). Most of the pyrrole was removed using a rotary evaporator and the crude residue was subject to chromatography over silica gel using a mixture of *n*-hexane/DCM (1/2) as the eluent. The solvent was removed under reduced pressure yielding **4** as a white or light red solid. Yield: 1.4 g (75%). ^{1}H NMR (600 MHz, CD_2Cl_2) *δ* 8.38 (s, 1H), 8.30 (s, 1H), 7.97 – 7.85 (m, 4H), 7.53 (dd, *J* = 8.5, 5.1 Hz, 2H), 7.37 (dd, *J* = 15.6, 8.7 Hz, 2H), 7.12 – 7.07 (m, 4H), 7.06 – 7.01 (m, 4H), 6.91 (d, *J* = 5.0 Hz, 4H), 6.88 (d, *J* = 7.0 Hz, 4H), 6.73 – 6.65 (m, 4H), 6.17 – 6.12 (m, 4H), 6.11 (s, 2H), 5.90 (d, *J* = 5.7 Hz, 2H), 5.83 – 5.74 (m, 4H), 2.33 (s, 6H), 2.29 (s, 6H), 2.06 (s, 12H), 2.02 (d, *J* = 11.5 Hz, 12H); ^{13}C NMR (150 MHz, CD_2Cl_2) δ 140.9, 140.8, 140.3, 140.1, 137.9, 137.9, 137.9, 136.9, 136.9, 136.8, 136.7, 136.6, 136.6, 136.5, 136.5, 136.3, 133.1, 132.7, 132.6, 132.5, 131.5, 131.0, 130.9, 130.7, 130.6, 130.3, 130.2, 128.4, 128.2, 128.1, 128.1, 128.0, 127.9, 122.3, 122.3, 116.9, 116.8, 116.8, 108.8, 108.7, 108.6, 108.6, 107.9, 107.8, 107.7, 107.7, 45.5, 45.3, 44.9, 44.8, 21.5, 21.5, 21.3, 21.2, 21.0, 21.0, 20.9, 20.9; HRMS (ESI$^+$) *m/z* $[M+H]^+$: Calcd for $C_{82}H_{79}N_4^+$ 1119.6299, found: 1119.6296.

Synthesis of compound 5

Compound **4** (560 mg, 0.5 mmol) and pentafluorobenzaldehyde (215 mg, 1.1 mmol) were dissolved in 500 mL dry dichloromethane and stirred fully for 10 minutes under nitrogen atmosphere. Boron trifluoride diethyl ether (130 uL) was slowly added into the reaction bottle with protection from ambient light. The reaction was maintained at room temperature for 2.5 h. Then, DDQ (680 mg, 3.0 mmol) was added and the reaction allowed to continue for 0.5 hours exposed to the air. Triethylamine (0.5 mL) was then added and the resulting black residue was first subjected to a flash chromatography over Alumina-B using DCM as the eluent. The desired product was collected as the first black-green band. This crude material was subject to a second round of silica gel chromatography (PE/DCM:3/1, eluent) before the product was separated by recycling GPC (DCM, eluent). Compound **5** was obtained as a black-green solid. Yield: 41 mg (6%).^{1}H NMR (600 MHz, CD_2Cl_2) *δ* 17.83 (s, NH), 16.99 (s, 2H), 12.30 (s, NH), 7.74 (d, *J* = 8.3 Hz, 4H), 7.02 (d, *J* = 8.3 Hz, 4H), 6.84 (s, 4H), 6.80 (s, 4H), 6.58 (d, *J* = 8.7 Hz, 2H), 6.19 (d, *J* = 5.2 Hz, 2H), 5.87 (d, *J* = 5.2 Hz, 2H), 5.68 (d, *J* = 8.8 Hz, 2H), 5.49 (d, *J* = 5.3 Hz, 2H), 5.15 (d, *J* = 5.2 Hz, 2H), 2.25 (s, 6H), 2.21 (s, 6H), 2.17 (s, 12H), 2.08 (s, 12H); ^{13}C NMR (150 MHz, CD_2Cl_2) δ 138.6, 138.1, 137.9, 137.7, 137.5, 137.4, 137.4, 137.2, 137.0, 135.5, 135.4, 134.2, 134.1, 132.1, 131.8, 131.6, 128.7, 128.5, 128.1, 127.5, 126.5, 126.5, 100.4, 21.2, 21.1, 20.7, 19.9; ^{19}F NMR (565 MHz, CD_2Cl_2) *δ* -139.30 (dd, *J* = 63.7, 22.8 Hz, 4F), -155.79 (t, *J* = 20.5 Hz, 1F), -156.05 (t, *J* = 19.9 Hz, 1F), -162.28 (t, *J* = 21.9 Hz, 2F), -162.69 (t, *J* = 22.0 Hz, 2F); HRMS (MALDI-TOF):*m/z* $[M]^+$: Calcd for $C_{96}H_{68}F_{10}N_4^+$ 1466.5279; found: 1466.5362.

Synthesis of compound 6

Compound **5** (60 mg, 0.04 mmol) was dissolved in 15 mL dry toluene under a nitrogen atmosphere. Then, ethyldiisopropylamine (0.9 mL) was added into the reaction flask and the mixture stirred for 10 min at room temperature. Boron trifluoride diethyl ether (1 mL) was slowly added and the mixture allowed to react at 80 ℃ for 12 h. The crude product was concentrated and dried by under reduced pressure before extraction with DCM and water. The organic phases were collected and purified by silica gel column chromatography (PE/DCM:3/1, eluent). Compound **6** was obtained as a dark blue solid. Yield: 44 mg (70%). ^{1}H NMR (400 MHz, CD_2Cl_2) δ 17.89 (s, NH), 17.04 (s, 2H), 6.99 (d, J = 7.9 Hz, 4H), 6.86 – 6.78 (m, 12H), 6.55 (d, J = 5.3 Hz, 2H), 6.45 (d, J = 8.7 Hz, 2H), 6.21 (d, J = 4.6 Hz, 2H), 5.72 (d, J = 8.9 Hz, 2H), 5.48 (d, J = 5.2 Hz, 2H), 5.15 (d, J = 4.9 Hz, 2H), 2.23 (d, J = 8.8 Hz, 12H), 2.18 (s, 12H), 2.08 (s, 12H); ^{13}C NMR (100 MHz, CD_2Cl_2) δ 160.0, 146.8, 142.2, 140.8, 139.4, 138.5, 138.3, 138.0, 137.7, 137.7, 137.3, 137.2, 137.1, 134.7, 134.1, 133.0, 129.6, 129.0, 128.9, 128.4, 127.9, 126.5, 123.5, 100.4, 21.1, 21.0, 19.9; ^{19}F NMR (376 MHz, CD_2Cl_2) δ -123.22 (dd, J = 60.2, 29.8 Hz, 2F), -138.25 (d, J = 21.1 Hz, 2F), -139.35 (d, J = 21.5 Hz, 2F), -154.76 (t, J = 20.2 Hz, 1F), -155.84 (t, J = 20.3 Hz, 1F), -162.02 – -162.41 (m, 4F); HRMS (ESI^+):*m/z* $[M+H]^+$: Calcd for $C_{96}H_{68}BF_{12}N_4^+$ 1514.5376; found: 1514.5364.

Synthesis of compound 7

Compound **6** (20 mg, 0.013 mmol) and $Cu(OAc)_2$ (40 mg, 0.22 mmol) were added to a 25 mL round-bottom flask containing a mixed solvent ($CHCl_3/CH_3CN$:6 mL/4 mL). The temperature was raised and the reaction mixture held at reflux for 48 h with stirring. After the volatiles were removed under reduced pressure, the residue was purified by silica gel column chromatography using PE/DCM:4/1 as the eluent. The product was obtained as a dark blue solid. Yield: 5 mg (24%).^{1}H NMR (600 MHz, CD_2Cl_2) δ 7.00 (d, J = 7.8 Hz, 4H), 6.85 – 6.82 (m, 8H), 6.81 (d, J = 8.1 Hz, 4H), 6.53 (d, J = 5.3 Hz, 2H), 6.22 (s, 1H), 6.21 (s, 1H), 6.19 (d, J = 5.3 Hz, 2H), 5.69 (d, J = 8.5 Hz, 2H), 5.65 (d, J = 5.2 Hz, 2H), 5.46 (d, J = 5.1 Hz, 2H), 2.24 (s, 12H), 2.14 (s, 12H), 2.07 (s, 12H); ^{13}C NMR (150 MHz, CD_2Cl_2) δ 160.1, 158.0, 149.9, 146.7, 142.2, 142.1, 139.4, 138.3, 137.8, 137.3, 137.3, 136.6, 135.2, 135.0, 134.6, 133.4, 129.7, 128.9, 128.3, 126.0, 125.2, 123.5, 21.1, 21.1, 21.0, 20.0; ^{19}F NMR (565 MHz, CD_2Cl_2) δ -122.96 (dd, J = 60.3, 29.9 Hz, 2F), -138.25 (d, J = 22.2 Hz, 2F), -139.26 (d, J = 23.5 Hz, 2F), -154.78 (t, J = 21.2 Hz, 1F), -155.23 (d, J = 23.2 Hz, 1F), -161.93 – -162.34 (m, 4F); HRMS (ESI^+):*m/z* $[M]^+$: Calcd for $C_{96}H_{64}BCuF_{12}N_4^+$ 1574.4337; found: 1574.4235.

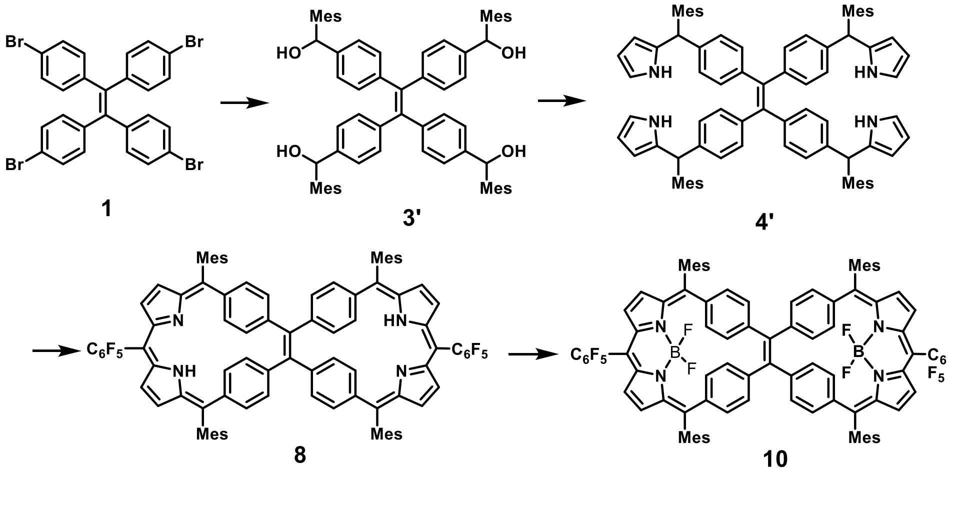

Supplementary Figure 2. Synthetic routes to **8** and **10**.

Synthesis of compound 3'

Compound **1** (3.0 g, 4.6 mmol) was dissolved in 200 mL dry THF under a nitrogen atmosphere and stirred in a low-temperature reactor at -78 °C for 10 minutes. Then, 18.5 mL n-butyl lithium (2.5 M in hexane) was slowly added. 1h later, 13.9 mL mesitylaldehyde were added. After one hour, the reaction was allowed to warm to room temperature, and 10 mL water were added to quench the reaction. The insoluble solids were removed by filtration. The filtrate was collected and the volatiles removed using a rotary evaporator. Further purification by column chromatography over silica gel (PE/EA: 4/1 as the eluent) gave 1.4 g of **3'** as a white solid. Yield: 32%. ^{1}H NMR (600 MHz, $CDCl_3$) δ 6.97 – 6.94 (m, 8H), 6.92 – 6.90 (m, 8H), 6.82 (d, J = 13.1 Hz, 8H), 6.21 (s, 4H), 2.28 (s, 12H), 2.17 (s, 12H), 2.15 – 2.12 (m, 12H); ^{13}C NMR (150 MHz, $CDCl_3$) δ 142.1, 142.1, 142.1, 142.0, 141.5, 141.5, 141.4, 141.3, 137.3, 137.1, 137.1, 136.8, 136.7, 136.7, 131.2, 131.2, 131.2, 130.1, 130.1, 124.8, 124.8, 124.7, 100.0, 71.0, 70.9, 21.0, 21.0, 20.6, 20.6; HRMS (ESI$^+$) m/z $[M+NH_4]^+$: Calcd for $C_{66}H_{72}NO_4^+$ 942.5456, found: 942.5459.

Synthesis of compound 4'

In a 100 mL round-bottomed flask equipped with a reflux condenser and a magnetic stirrer, precursor **3'** (1.5 g, 1.6 mmol) was added along with dry pyrrole (20 mL) After dissolution, the solution was purged with nitrogen for 20 minutes. Subsequently, boron trifluoride diethyl etherate (0.5 mL) was added and the solution was heated at reflux for 20 h under a nitrogen atmosphere. The solution was neutralized via the addition of triethylamine (1.5 mL). Most of the pyrrole was removed using a rotary evaporator and the crude residue was subject to chromatography over silica gel using a mixture of *n*-hexane/DCM (1/2) as the eluent. The solvent was removed under reduced pressure yielding **4'** as a white or light red solid. Yield: 1.2 g (67%); ^{1}H NMR (600 MHz, CD_2Cl_2) δ 7.87 (d, J = 8.7 Hz, 4H), 6.96 – 6.92 (m, 8H), 6.89 – 6.86 (m, 8H), 6.85 (s, 8H), 6.66 (s, 4H), 6.13 – 6.09 (m, 4H), 5.82 (s, 4H), 5.74 (d, J = 9.2 Hz, 4H), 2.28 (d, J = 3.4 Hz, 12H), 1.97 (s, 24H); ^{13}C NMR (150 MHz, CD_2Cl_2) δ 141.8, 141.7, 140.9, 140.9, 140.7, 140.6, 140.5, 137.4, 137.4, 136.2, 136.2, 132.1, 132.0, 131.8, 131.8, 131.2, 130.2, 127.7, 127.7, 116.4, 116.4, 108.2, 107.5, 107.4, 100.0, 44.4, 44.4, 21.1, 21.0, 20.5; HRMS (ESI$^+$) m/z $[M+H]^+$: Calcd for $C_{82}H_{81}N_4^+$ 1121.6456, found: 1121.6453.

Synthesis of compound 8

Compound **4'** (560 mg, 0.5 mmol) and pentafluorobenzaldehyde (215 mg, 1.1 mmol) were dissolved in 500 mL dry dichloromethane and stirred vigorously for 10 minutes under a nitrogen atmosphere. Boron trifluoride diethyl ether (130 uL) was slowly added into the reaction bottle with protection from ambient light. The reaction vessel was maintained at room temperature for 2.5 h. Then, DDQ (680 mg, 3.0 mmol) was added and the reaction allowed to continue for 0.5 hours with exposure to the laboratory atmosphere. Triethylamine (0.5 mL) was then added and the resulting black residue was first subjected to a flash chromatography over Alumina-B using DCM as the eluent. The desired product was collected as the first black-green band. This crude material was subject to a second round of silica gel chromatography (PE/DCM:3/1, eluent) before the product was separated by recycling GPC (DCM, eluent). Compound **8** was obtained as a black-green solid. Yield: 55 mg (8%). ^{1}H NMR (600 MHz, CD_2Cl_2) δ 13.44 (s, 2H), 7.95 (s, 8H), 7.14 (s, 8H), 6.84 (s, 8H), 5.98 (d, J = 5.2 Hz, 4H), 5.63 (d, J = 5.2 Hz, 4H), 2.24 (s, 12H), 2.12 (s, 24H); ^{13}C NMR (150 MHz, CD_2Cl_2) δ 141.3, 137.8, 137.8, 137.3, 136.8, 134.2, 132.7, 132.2, 128.6, 127.3, 21.2, 20.5; ^{19}F NMR (565 MHz, CD_2Cl_2) δ -139.16 – -139.75 (m, 4F), -156.09 (t, J = 20.1 Hz, 2F), -162.61

(t, *J* = 21.6 Hz, 4F); HRMS (ESI^+):*m/z* $[M+H]^+$: Calcd for $C_{96}H_{71}F_{10}N_4^+$ 1470.5548; found: 1470.5510.

Synthesis of compound 10

Compound **8** (60 mg, 0.04 mmol) was dissolved in 15 mL dry toluene under a nitrogen atmosphere. Ethyldiisopropylamine (0.9 mL) was then added into the reaction flask. The resulting mixture was stirred for 10 min at room temperature. Boron trifluoride diethyl ether (1 mL) was slowly added and the mixture allowed to react at 80 ℃ for 12 h. The crude product obtained in this way was concentrated and dried by under reduced pressure before being partitioned between DCM and water. After further DCM extractions, the oirganic phases were collected and purified by silica gel column chromatography (PE/DCM:3/1, eluent) giving **10** as a dark blue solid. Yield: 38 mg (59%). ^1H NMR (400 MHz, CD_2Cl_2) *δ* 7.17 (s, 16H), 6.82 (s, 8H), 6.50 (d, *J* = 6.4 Hz, 4H), 6.13 (d, *J* = 4.6 Hz, 4H), 2.22 (s, 12H), 2.10 (s, 24H); ^{13}C NMR (100 MHz, CD_2Cl_2) δ 160.4, 146.9, 146.6, 142.5, 142.3, 139.5, 138.2, 137.3, 137.0, 130.3, 128.8, 128.6, 123.3, 21.1, 20.9; ^{19}F NMR (376 MHz, CD_2Cl_2) *δ* -120.41 (dd, *J* = 61.0, 30.0 Hz, 4F), -138.27 (d, *J* = 21.5 Hz, 4F), -154.85 (t, *J* = 20.0 Hz, 2F), -162.11 (t, *J* = 20.4 Hz, 4F). HRMS (MALDI-TOF):*m/z* $[M]^+$: Calcd for $C_{96}H_{68}B_2F_{14}N_4^+$ 1562.5474; found: 1562.5541.

2. Photophysical measurements

Femtosecond transient absorption data were obtained using a lab-built femtosecond absorption spectrometer, which consisted of optical parametric amplifiers (Palitra, Quantronix) pumped by a Ti:sapphire regenerative amplifier system (Integra-C, Quantronix) operating at 1 kHz repetition rate and an optical detection system. The generated OPA pulses had a pulse width of ~ 100 fs and an average power of 100 mW in the range 280-2700 nm. These were used as pump pulses. White light continuum (WLC) probe pulses were generated using a sapphire window (3 mm of thickness) by focusing a small portion of the fundamental 800 nm pulses, which were picked off by a quartz plate before entering to the optical parametric amplifiers. After the measurements, the absorption and emission spectra were carefully checked to detect if there were artifacts due to degradation and photo-oxidation of samples (Z-202308028841 at the Research Support Center for Bio-Bigdata Analysis and Utilization of Biological Resources).

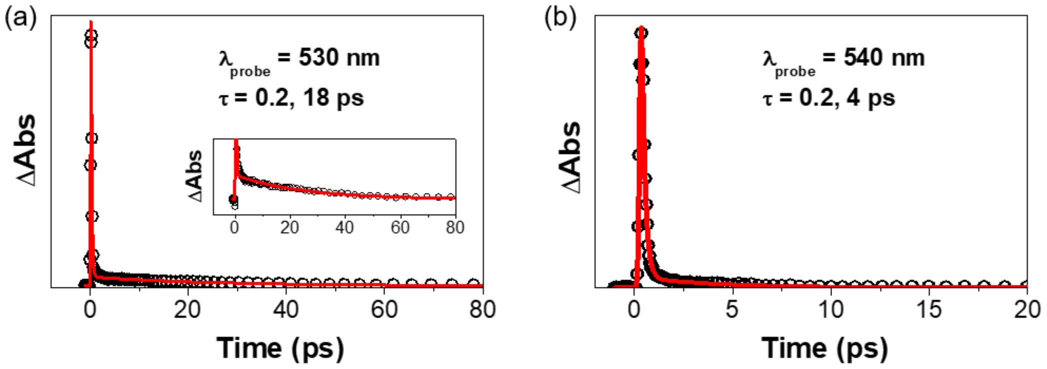

Supplementary Figure 3. Full TA decay profiles of (a) **5** and (b) **8**. Inset is an enlarged decay profile of **5**.

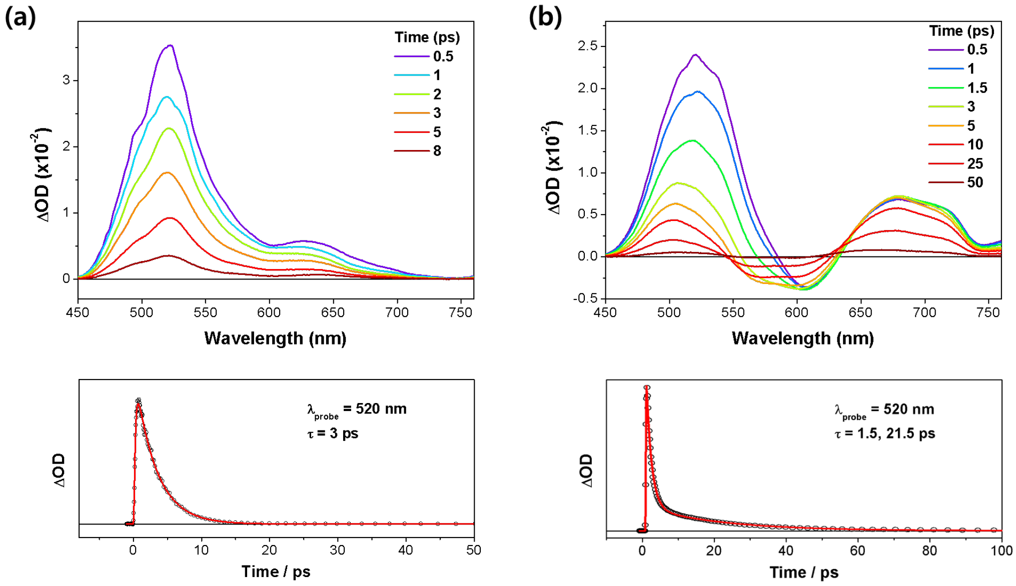

Supplementary Figure 4. TA spectra (top) and decay profiles (bottom) of (a) **7** and (b) **10** in toluene with photoexcitation at 600 nm.

3. Electrochemistry

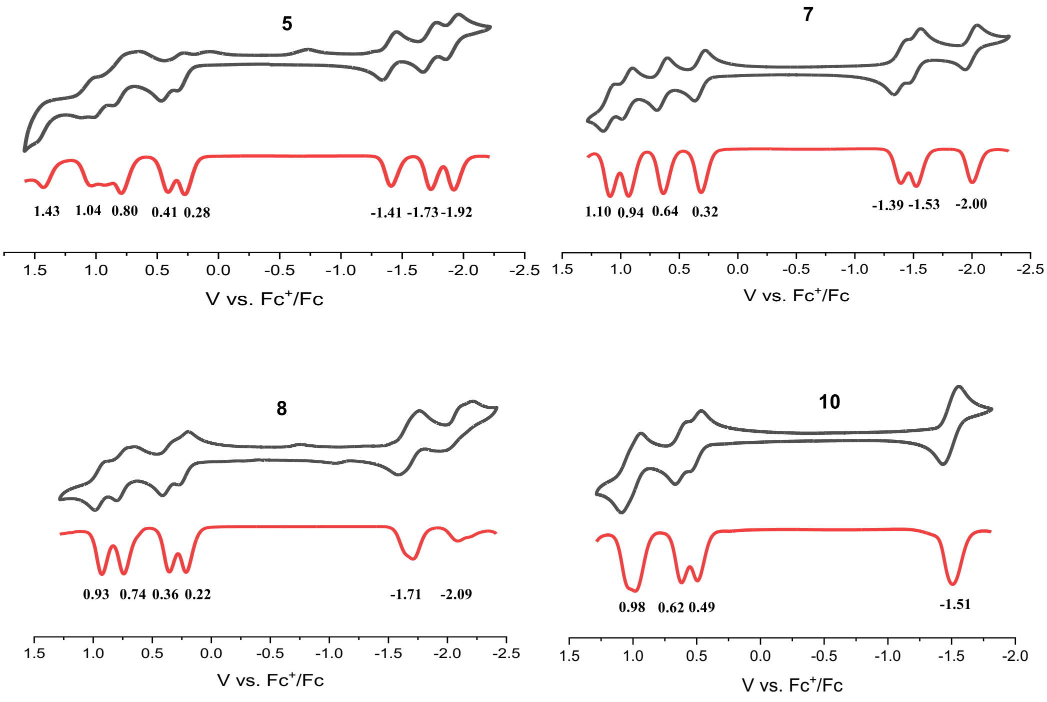

Supplementary Figure 5. CV and DPV curves for **5**, **7**, **8** and **10** recorded in dry DCM containing 0.1 M $TBAPF_6$. The scan rate is 50 $mV \cdot s^{-1}$.

4. Quantum chemical calculations

Quantum mechanical calculations were carried out with Gaussian 16 program suite.[1] All structural optimizations were made and comparatively analyzed using density functional theory (DFT) with B3LYP employing the 6-31G(d,p) basis set.[2] The X-ray crystallographic structures were used as initial geometries for geometry optimization.

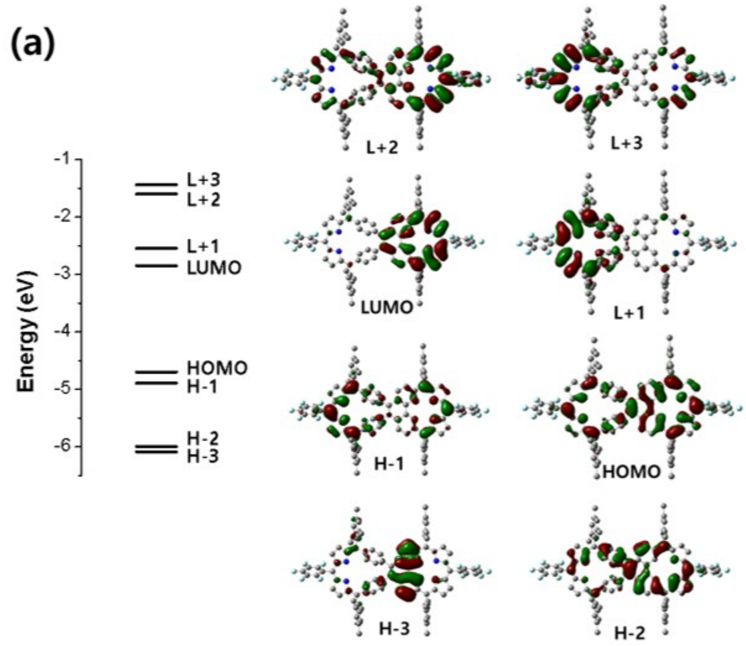

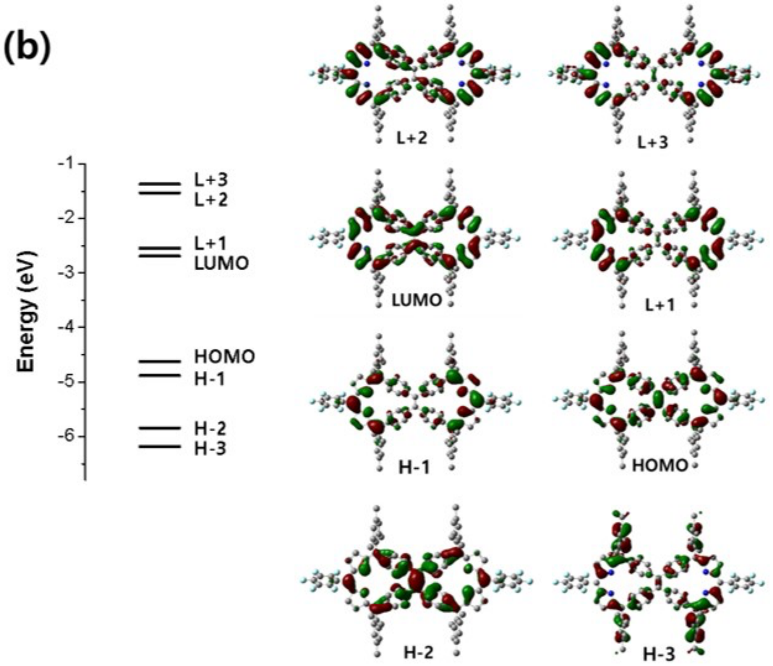

Supplementary Figure 6. Energy diagrams and frontier molecular orbitals for (a) **5** and (b) **8**.

Supplementary Table 1. Simulated electronic absorption transitions and MO contributions to **5** and **8**.

**5**			
No.	Wavelength (nm)	Osc. Strength	Major Molecular Orbital Contributions
1	911.5752	0.0383	HOMO->LUMO (90%)
2	721.8406	0.0048	H-1->LUMO (78%), HOMO->L+1 (13%)
3	680.0688	0.0435	H-1->LUMO (13%), HOMO->L+1 (85%)
4	592.5696	0.1839	H-1->L+1 (96%)
5	466.0852	0.0737	H-2->LUMO (36%), HOMO->L+2 (46%)
6	459.7254	0.0339	H-3->LUMO (75%)
7	438.6151	0.4209	H-5->LUMO (12%), H-2->LUMO (41%), HOMO->L+2 (28%)
8	434.8919	0.0506	H-5->LUMO (15%), H-2->L+1 (10%), H-1->L+2 (17%), HOMO->L+3 (35%)
9	426.8664	0.064	H-7->LUMO (12%), H-6->LUMO (16%), H-5->LUMO (46%)
10	412.1923	0.0272	H-1->L+2 (51%), HOMO->L+3 (43%)
11	409.9165	0.1903	H-10->LUMO (20%), H-7->LUMO (36%)
12	407.8399	0.1264	H-10->LUMO (19%), H-4->LUMO (41%)

**8**			
No.	Wavelength (nm)	Osc. Strength	Major contribs
1	832.3823	0.0739	HOMO->LUMO (96%)
2	741.7489	0.0019	H-1->LUMO (13%), HOMO->L+1 (86%)
3	657.1439	0.0008	H-1->LUMO (86%), HOMO->L+1 (13%)
4	594.2738	0.1967	H-1->L+1 (96%)
5	457.0477	0.2188	H-2->LUMO (34%), HOMO->L+2 (54%)
6	440.063	0.4453	H-2->LUMO (49%), HOMO->L+2 (30%), HOMO->L+4 (14%)
7	436.6841	0.0005	H-4->LUMO (11%), H-2->L+1 (31%), H-1->L+2 (15%), HOMO->L+3 (34%)
8	418.9192	0.0013	H-2->L+1 (58%), HOMO->L+3 (19%)
9	407.5316	0.0002	H-1->L+2 (51%), HOMO->L+3 (36%)
10	404.2495	0.41	H-4->L+1 (11%), H-3->LUMO (35%), H-1->L+3 (11%), HOMO->L+2 (12%), HOMO->L+4 (23%)
11	401.1886	0.5186	H-6->LUMO (12%), H-3->LUMO (18%), HOMO->L+4 (43%)
12	397.077	0.0021	H-6->L+1 (12%), H-5->LUMO (16%), H-4->LUMO (27%), H-1->L+2 (15%)

(a)

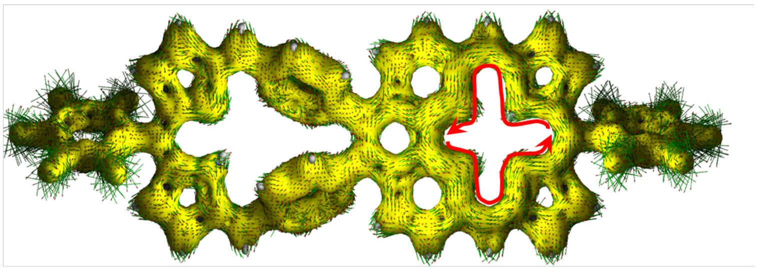

(b)

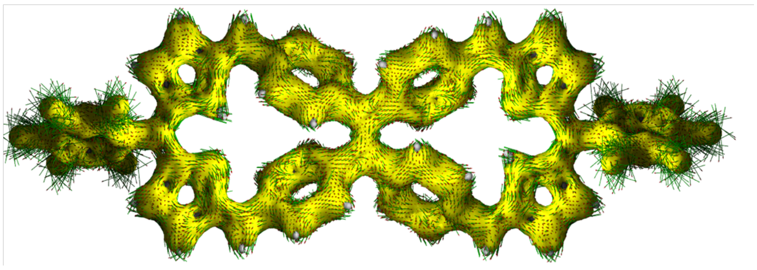

Supplementary Figure 7. ACID plots for (a) **5** and (b) **8** with an isovalue of 0.04. For clarity, mesityl substituents are omitted in the ACID calculations using their otherwise optimized structures.

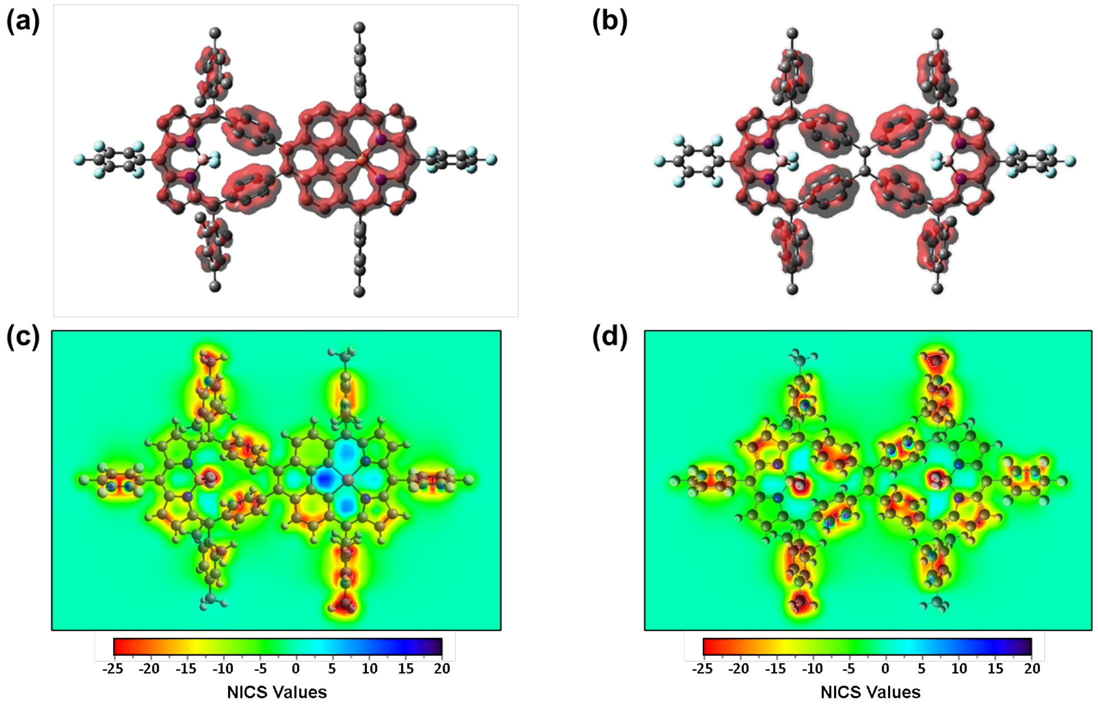

Supplementary Figure 8. EDDB plots of (a) **7** and (b) **10**, where the localized and delocalized cyclic p-conjugation pathways are visualized with red-colored surface with an isovalue of 0.014. NICS 2D maps of (c) **7** and (d) **10**, which are estimated 1.5 Å above their mean plane.

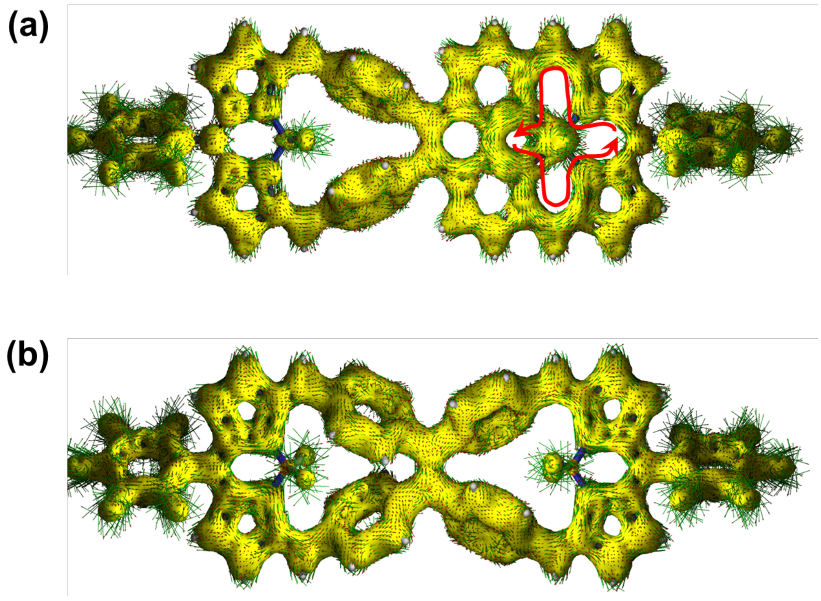

Supplementary Figure 9. The ACID plots of (a) **7** and (b) **10** with an isovalue of 0.04. For clarity, mesityl substituents are omitted in the ACID calculations using their otherwise optimized structures.

5. X-ray Crystallography

Supplementary Table 2. Crystal data and structure refinement for 5 (CCDC number: 2286144).

Empirical formula	$C_{96.57}$ $H_{69.15}$ $Cl_{1.15}$ F_{10} N_4	
Formula weight	1516.37	
Temperature	100.00(10) K	
Wavelength	1.54184 Å	
Crystal system	monoclinic	
Space group	$P2_1/c$	
Unit cell dimensions	a = 16.4820(2) Å	α= 90°.
	b = 13.59017(13) Å	β= 101.0420(11)°.
	c = 35.6020(4) Å	γ = 90°.
Volume	7826.96(15) $Å^3$	
Z	4	
Density (calculated)	1.287 g/cm^3	
Absorption coefficient	1.103 mm^{-1}	
F(000)	3145	
Crystal size	0.2 x 0.1 x 0.05 mm^3	
2θ range for data collection	8.128 to 152.684°	
Index ranges	-20<=h<=19, -17<=k<=12, -44<=l<=43	
Reflections collected	55556	
Independent reflections	15692 [R_{int} = 0.0492]	
Completeness to theta = 67.684°	99.4 %	
Max. and min. transmission	1.00000 and 0.94719	
Refinement method	Full-matrix least-squares on F^2	
Data / restraints / parameters	15692 / 239 / 1120	
Goodness-of-fit on F^2	1.165	
Final R indices [I>2sigma(I)]	R_1 = 0.0739, wR_2 = 0.1587	
R indices (all data)	R_1 = 0.0979, wR_2 = 0.1682	
Largest diff. peak and hole	0.270 and -0.232 e.$Å^{-3}$	

Supplementary Table 3. Crystal data and structure refinement for 7 (CCDC number: 2286143).

Empirical formula	C_{96} H_{64} B Cu F_{12} N_4	
Formula weight	1575.86	
Temperature	100.02(13) K	
Wavelength	1.54184 Å	
Crystal system	monoclinic	
Space group	$P2_1$	
Unit cell dimensions	a = 19.2663(5) Å	α= 90°.
	b = 12.3653(2) Å	β= 93.078(3)°.
	c = 20.4913(6) Å	γ = 90°.
Volume	4874.7(2) $Å^3$	
Z	2	
Density (calculated)	1.074 g/cm^3	
Absorption coefficient	0.856 mm^{-1}	
F(000)	1620	
Crystal size	0.2 x 0.2 x 0.1 mm^3	
2θ range for data collection	6.472 to 152.938°	
Index ranges	-23<=h<=24, -13<=k<=15, -23<=l<=25	
Reflections collected	38310	
Independent reflections	15170 [R_{int} = 0.0890]	
Completeness to theta = 67.684°	99.8 %	
Max. and min. transmission	1.00 and 0.447	
Refinement method	Full-matrix least-squares on F^2	
Data / restraints / parameters	15170 / 1407 / 1207	
Goodness-of-fit on F^2	1.022	
Final R indices [I>2sigma(I)]	R_1 = 0.0776, wR_2 = 0.2079	
R indices (all data)	R_1 = 0.0886, wR_2 = 0.2172	
Absolute structure parameter	0.33(5)	
Largest diff. peak and hole	0.645 and -0.501 e.$Å^{-3}$	

Supplementary Table 4. Crystal data and structure refinement for 8 (CCDC number: 2286146).

Empirical formula	$C_{55} H_{43} F_5 N_2$	
Formula weight	826.91	
Temperature	99.99(10) K	
Wavelength	1.54184 Å	
Crystal system	monoclinic	
Space group	P2/c	
Unit cell dimensions	a = 16.1332(2) Å	α= 90°.
	b = 11.10779(12) Å	β= 97.4243(12)°.
	c = 24.5852(3) Å	γ = 90°.
Volume	4368.85(9) $Å^3$	
Z	4	
Density (calculated)	1.257 g/cm^3	
Absorption coefficient	0.715 mm^{-1}	
F(000)	1728	
Crystal size	0.3 x 0.2 x 0.05 mm^3	
2θ range for data collection	7.252 to 152.922°	
Index ranges	-20<=h<=20, -11<=k<=14, -30<=l<=28	
Reflections collected	30195	
Independent reflections	8792 [R_{int} = 0.0516]	
Completeness to theta = 67.684°	99.9 %	
Max. and min. transmission	1.00000 and 0.50671	
Refinement method	Full-matrix least-squares on F^2	
Data / restraints / parameters	8792 / 408 / 677	
Goodness-of-fit on F^2	1.047	
Final R indices [I>2sigma(I)]	R_1 = 0.0594, wR_2 = 0.1601	
R indices (all data)	R_1 = 0.0695, wR_2 = 0.1665	
Largest diff. peak and hole	0.755 and -0.560 e.$Å^{-3}$	

Supplementary Table 5. Crystal data and structure refinement for 10 (CCDC number: 2286274).

Empirical formula	$C_{96.5} H_{70} B_2 F_{14} N_4 O_{0.5}$	
Formula weight	1581.18	
Temperature	100.00(10)K	
Wavelength	1.54184 Å	
Crystal system	monoclinic	
Space group	$P2_1/n$	
Unit cell dimensions	a = 18.3402(3) Å	α= 90°.
	b = 38.3048(4) Å	β= 106.5977(16)°.
	c = 25.4905(4) Å	γ = 90°.
Volume	17161.4(4) $Å^3$	
Z	8	
Density (calculated)	1.224 g/cm^3	
Absorption coefficient	0.775 mm^{-1}	
F(000)	6536.0	
Crystal size	0.7 x 0.5 x 0.2 mm^3	
2θ range for data collection	6.826 to 149.002°	
Index ranges	$-22 \leq h \leq 22$, $-45 \leq k \leq 47$, $-31 \leq l \leq 31$	
Reflections collected	251018	
Independent reflections	34859 [R_{int} = 0.1478]	
Completeness to theta = 67.684°	99.9 %	
Max. and min. transmission	1.00000 and 0.62425	
Refinement method	Full-matrix least-squares on F^2	
Data/restraints/parameters	34859/0/2134	
Goodness-of-fit on F^2	1.117	
Final R indexes [I>=2 sigma (I)]	R_1 = 0.0659, wR_2 = 0.1759	
Final R indexes [all data]	R_1 = 0.0877, wR_2 = 0.2024	
Largest diff. peak and hole	0.54 and -0.40e $Å^{-3}$	

6. ^{1}H, ^{13}C, ^{19}F, 2D-COSY NMR, HR-MALDI-TOF-MS and HR-ESI-MS spectra

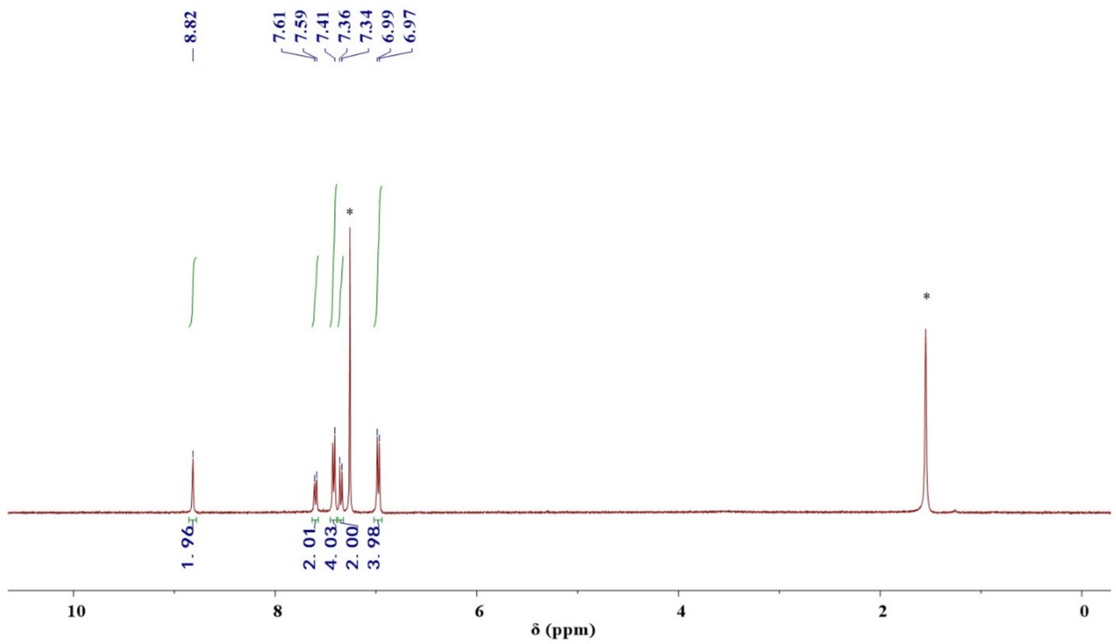

Supplementary Figure 10. ^{1}H NMR spectrum of **2** in $CDCl_3$. Asterisks indicate residual peaks arising from the solvent or impurities.

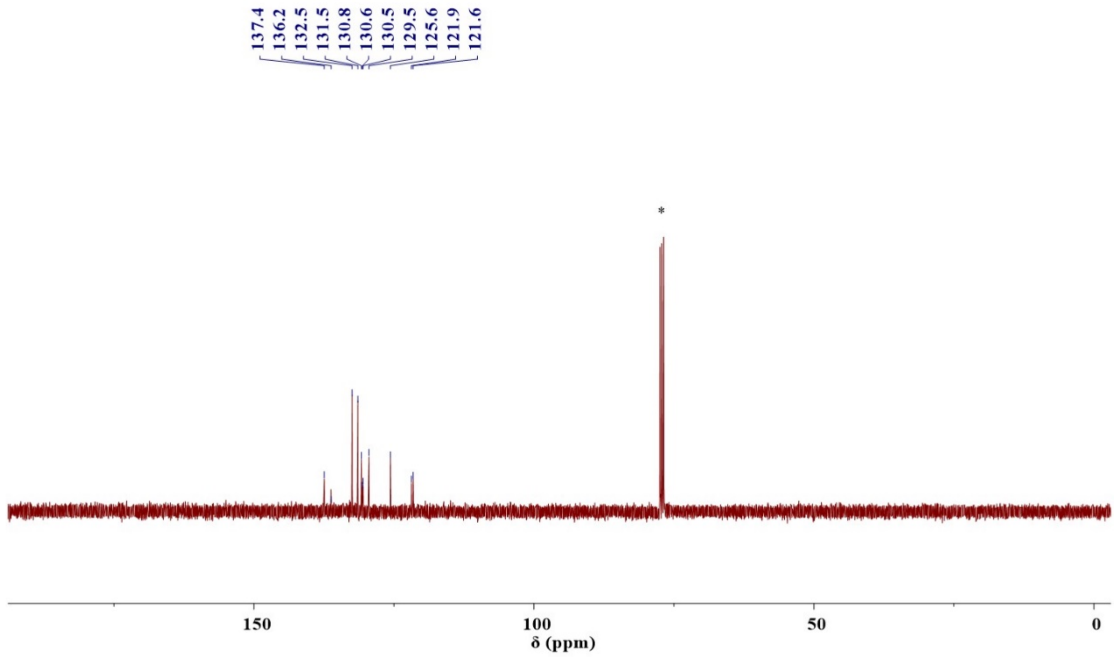

Supplementary Figure 11. ^{13}C NMR spectrum of **2** in $CDCl_3$. Asterisks indicate residual peaks arising from the solvent or impurities.

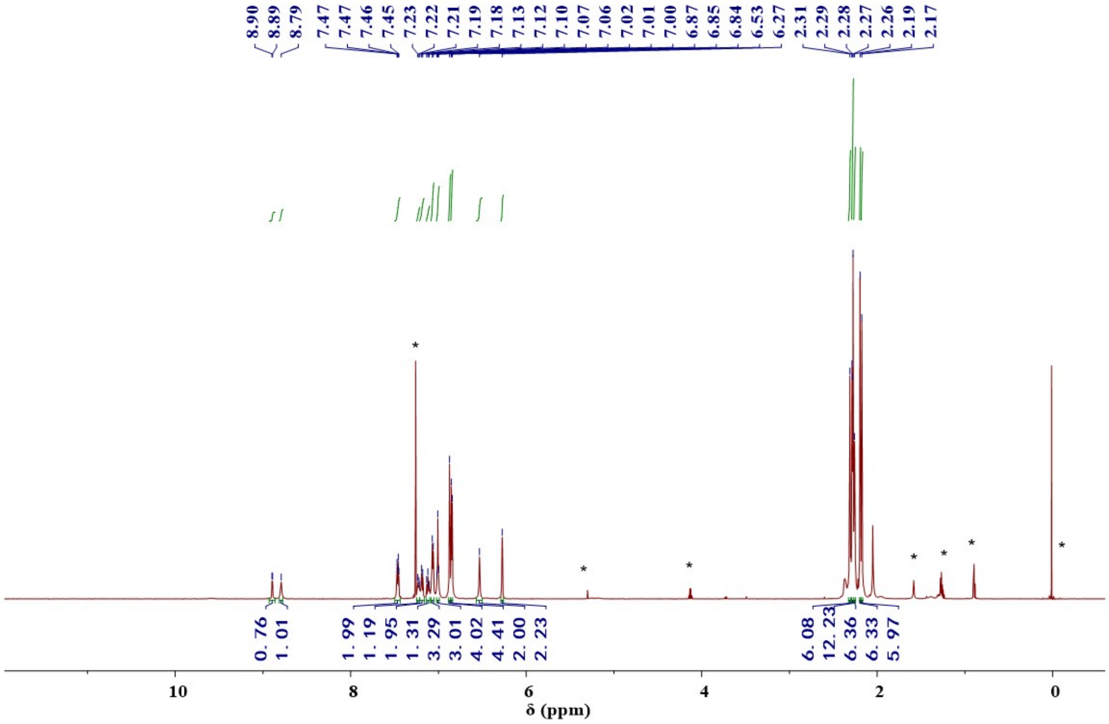

Supplementary Figure 12. ^{1}H NMR spectrum of **3** in $CDCl_3$. Asterisks indicate residual peaks arising from the solvent or impurities.

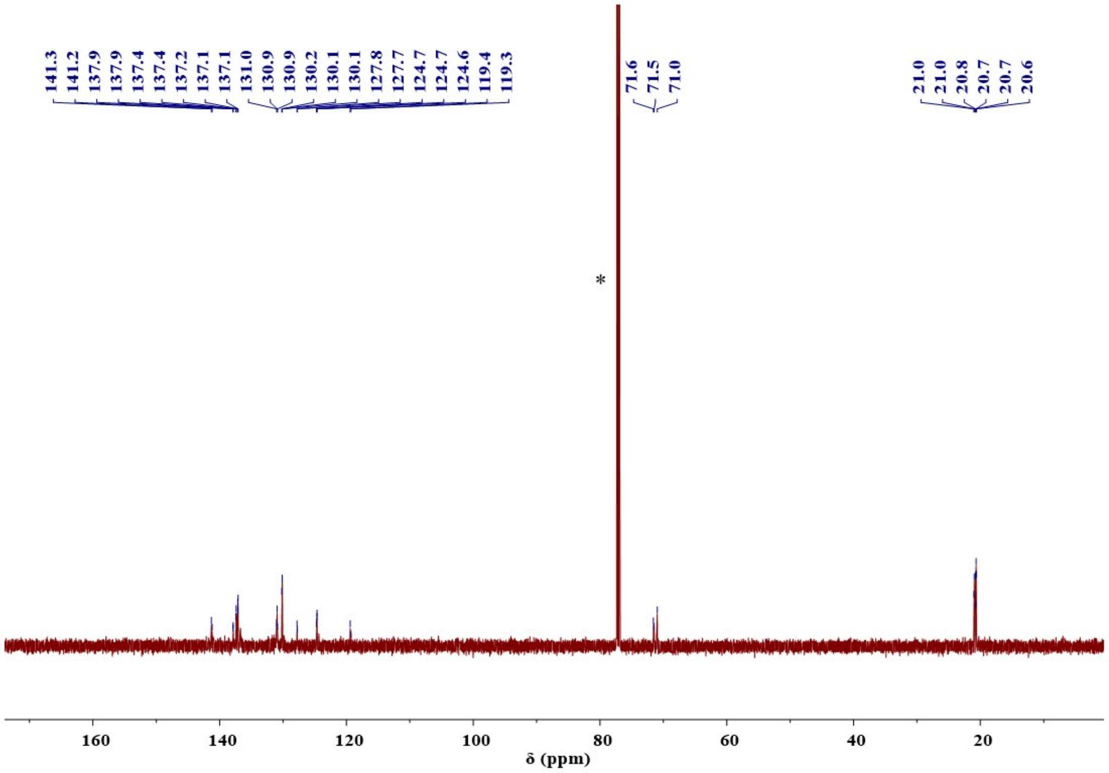

Supplementary Figure 13. ^{13}C NMR spectrum of **3** in $CDCl_3$. Asterisks indicate residual peaks arising from the solvent or impurities.

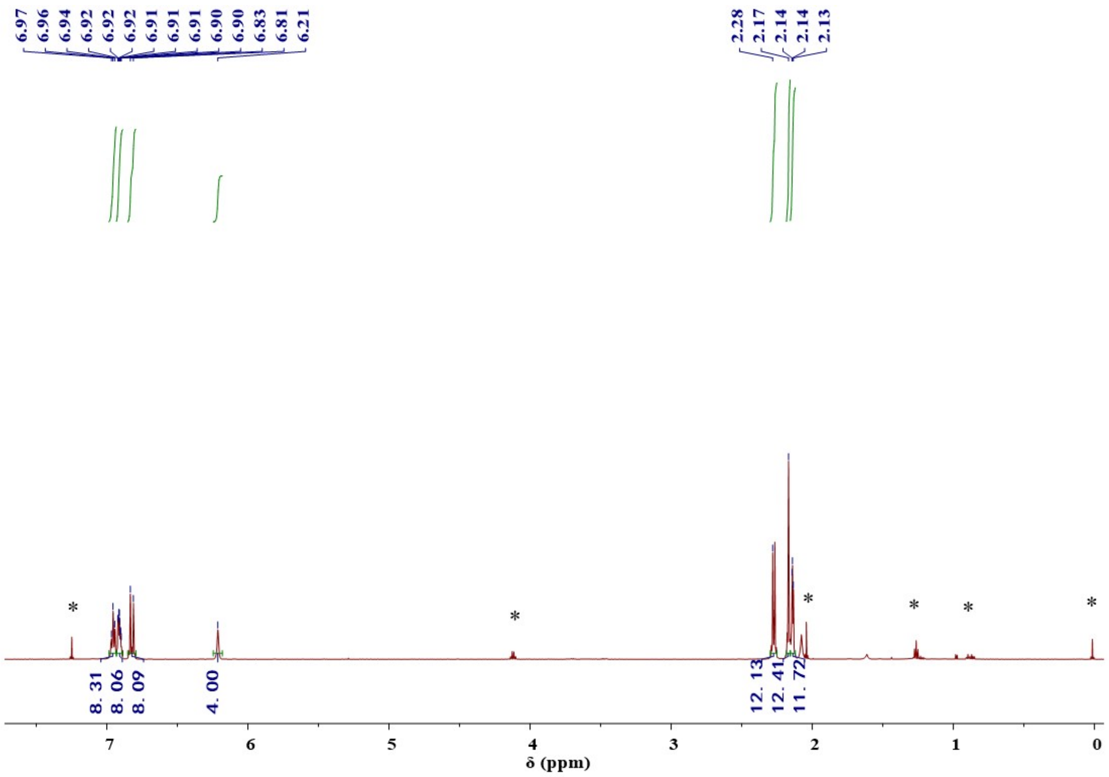

Supplementary Figure 14. ^{1}H NMR spectrum of **3'** in $CDCl_3$. Asterisks indicate residual peaks arising from the solvent or impurities.

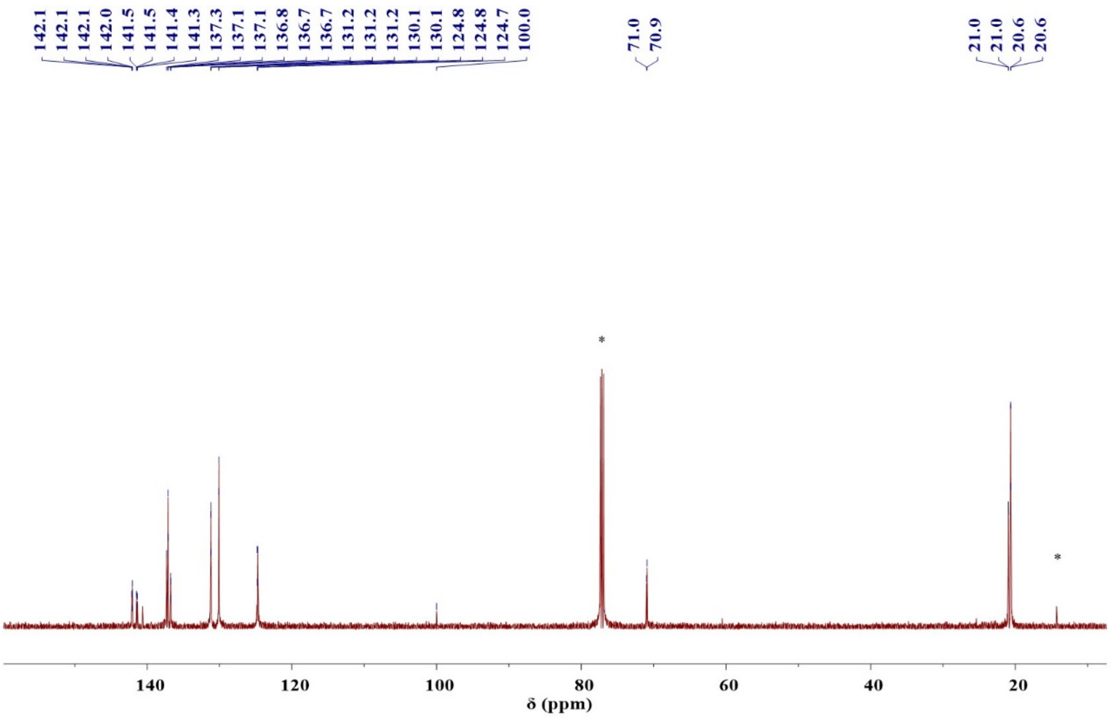

Supplementary Figure 15. ^{13}C NMR spectrum of **3'** in $CDCl_3$. Asterisks indicate residual peaks arising from the solvent or impurities.

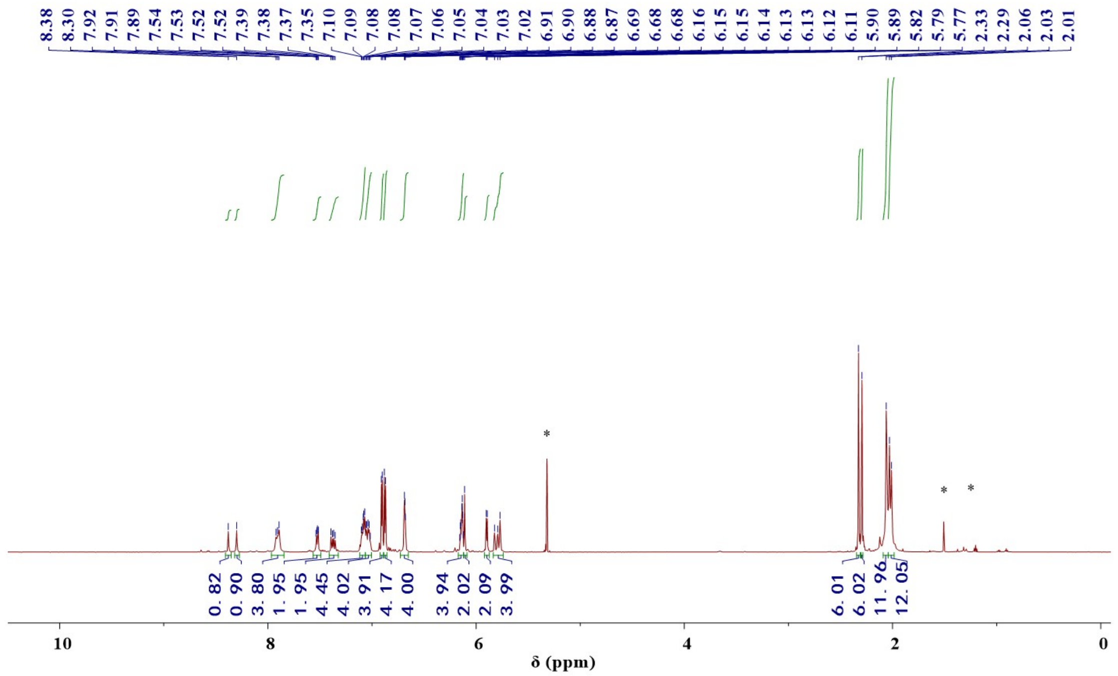

Supplementary Figure 16. ^{1}H NMR spectrum of **4** in CD_2Cl_2. Asterisks indicate residual peaks arising from the solvent or impurities.

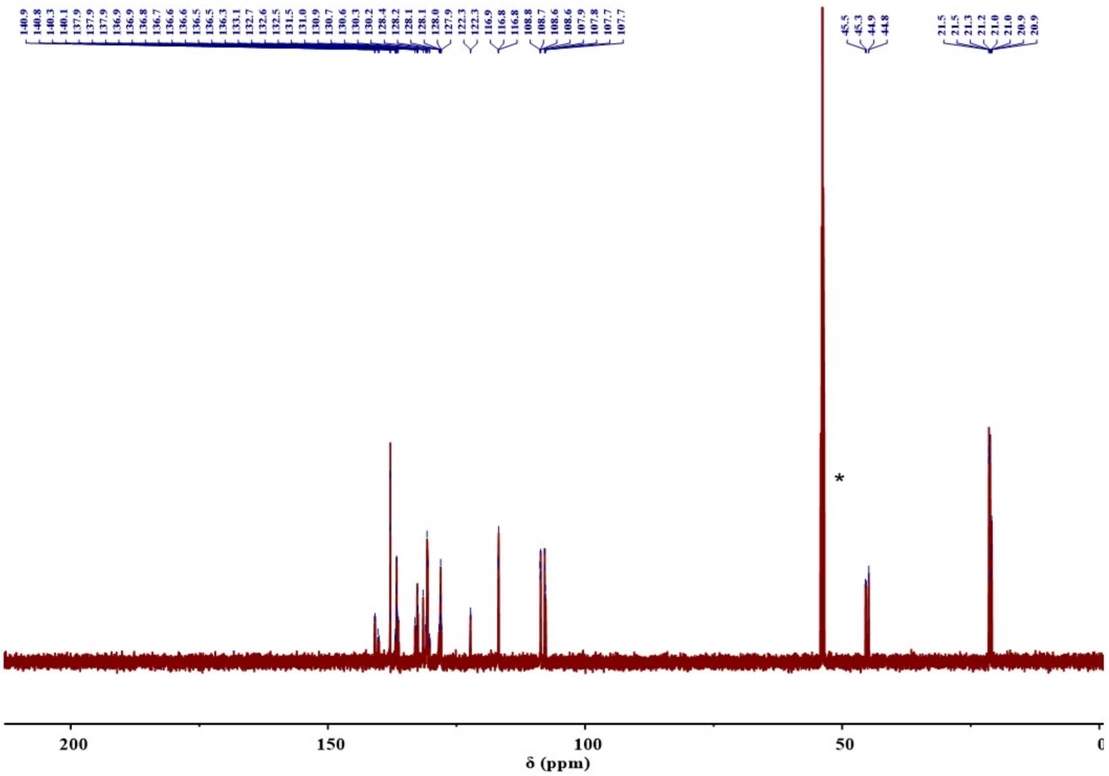

Supplementary Figure 17. ^{13}C NMR spectrum of **4** in CD_2Cl_2. Asterisks indicate residual peaks arising from the solvent or impurities.

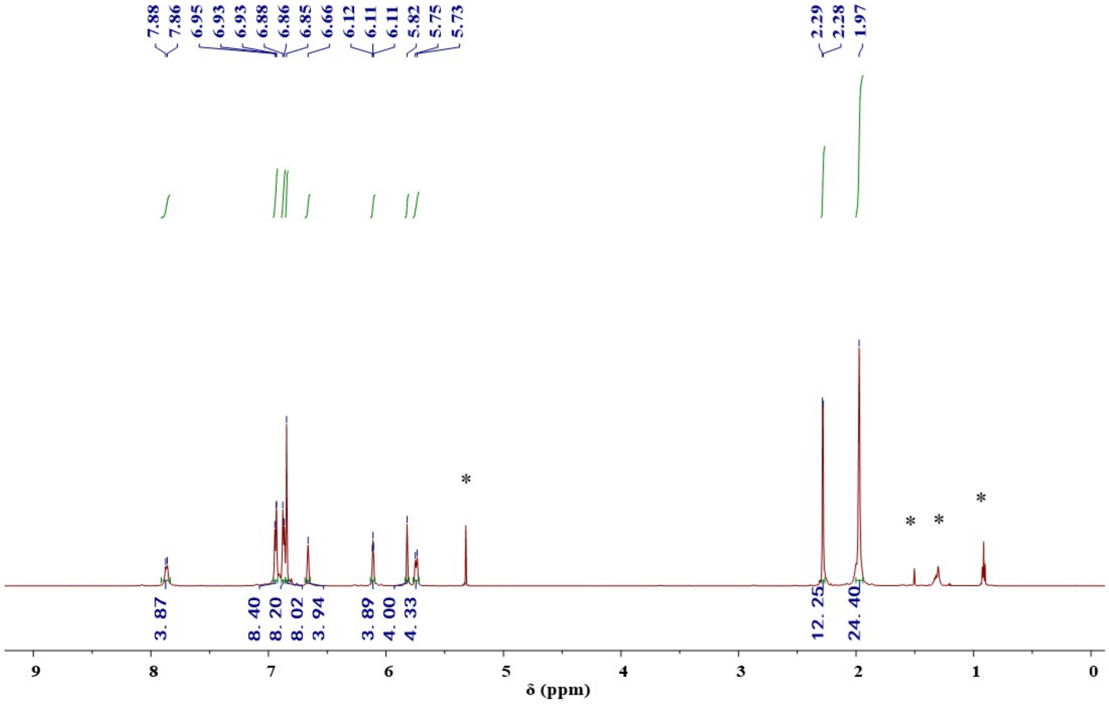

Supplementary Figure 18. ^{1}H NMR spectrum of **4'** in CD_2Cl_2. Asterisks indicate residual peaks arising from the solvent or impurities.

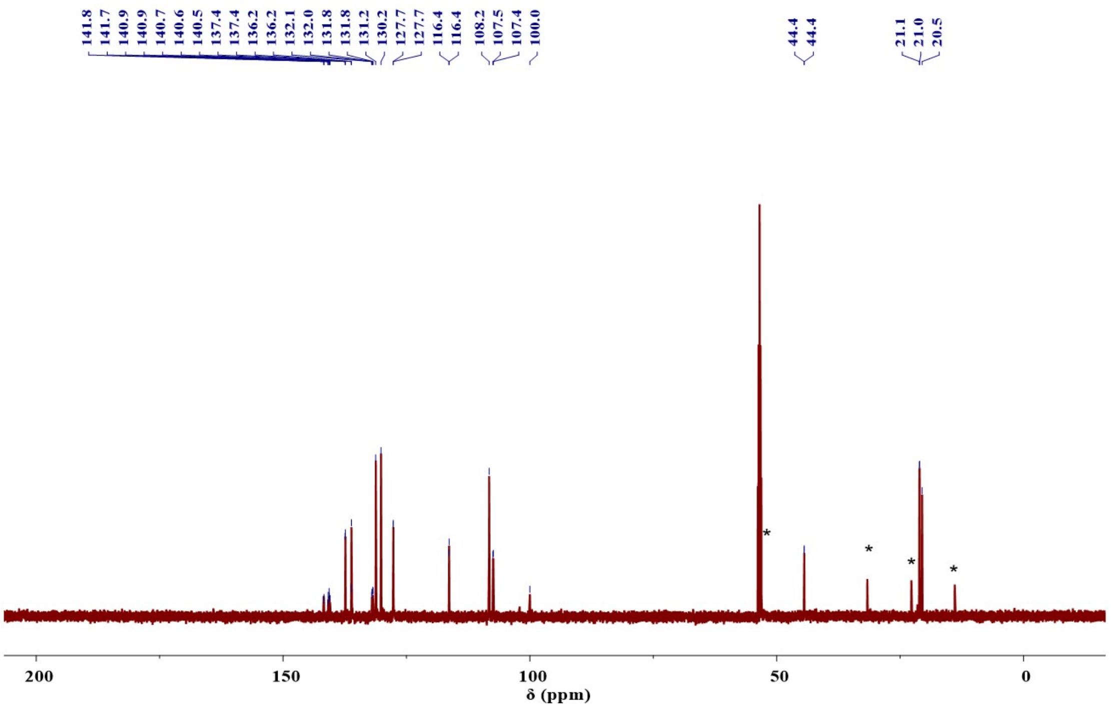

Supplementary Figure 19. ^{13}C NMR spectrum of **4'** in CD_2Cl_2. Asterisks indicate residual peaks arising from the solvent or impurities.

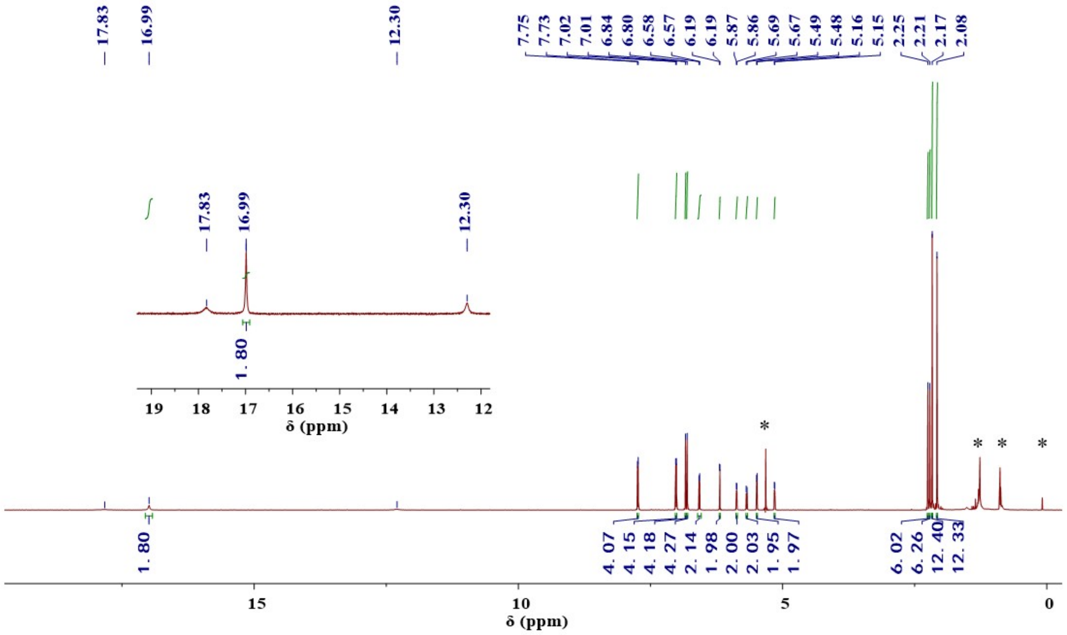

Supplementary Figure 20. ^{1}H NMR spectrum of **5** in CD_2Cl_2. Asterisks indicate residual peaks arising from the solvent or impurities.

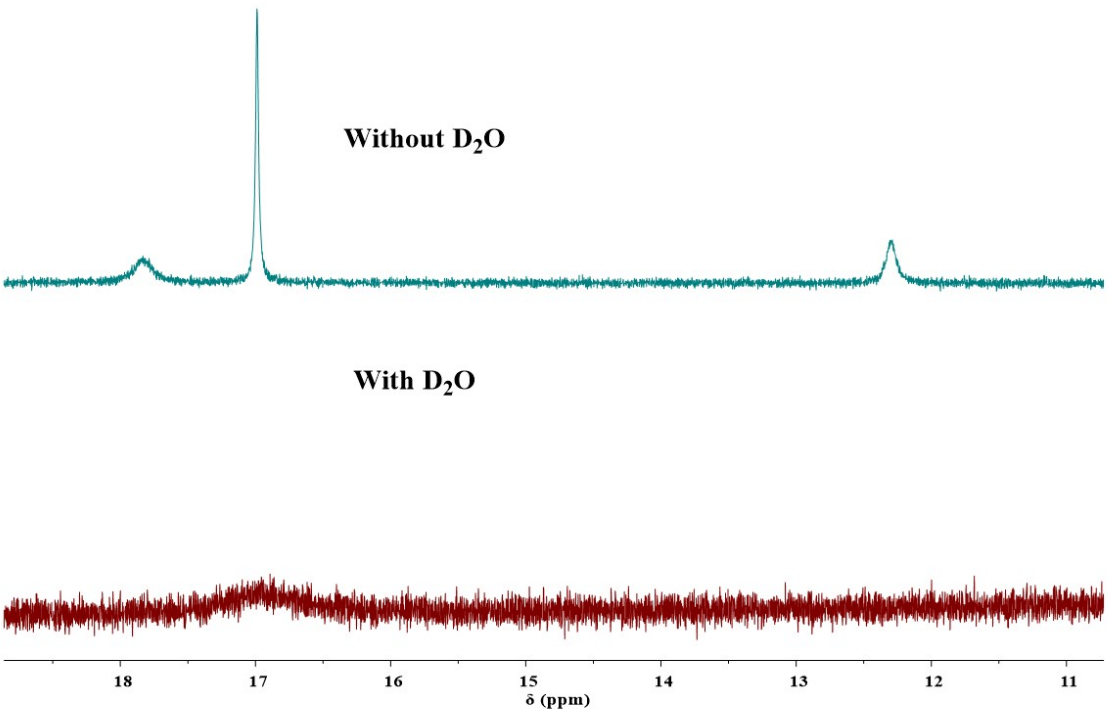

Supplementary Figure 21. Partial ^1H NMR spectrum of **5** without and with D_2O in CD_2Cl_2.

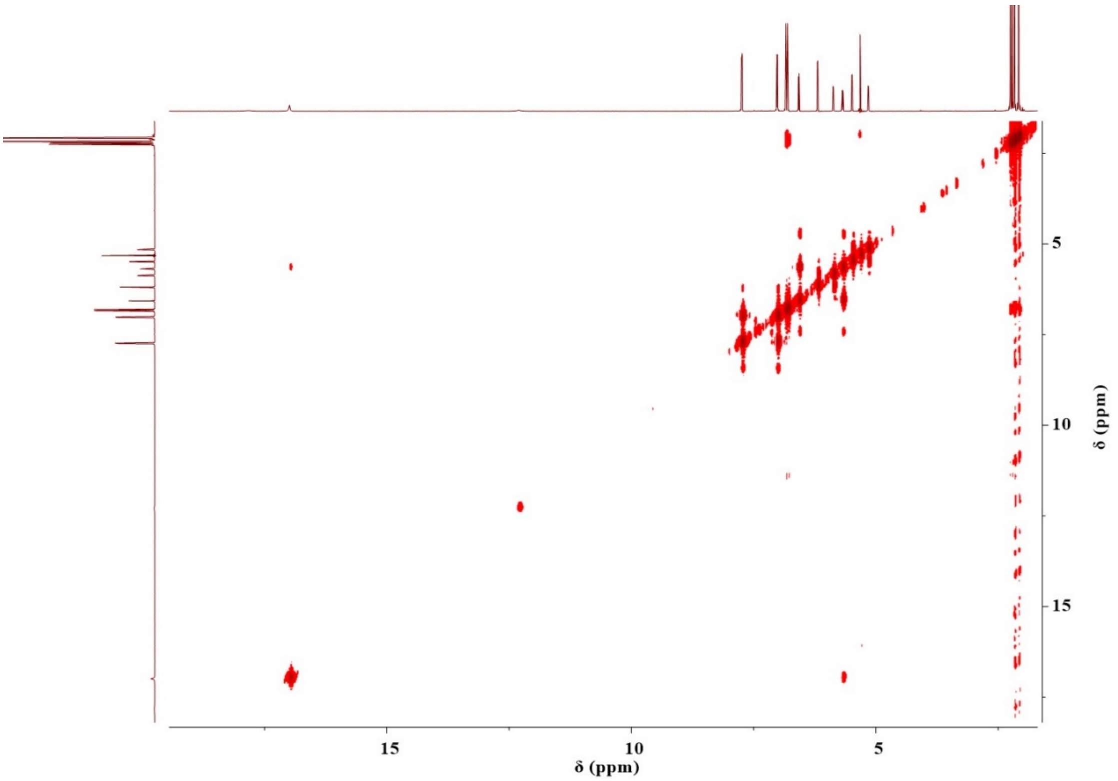

Supplementary Figure 22. 2D-COSY spectrum of **5** in CD_2Cl_2.

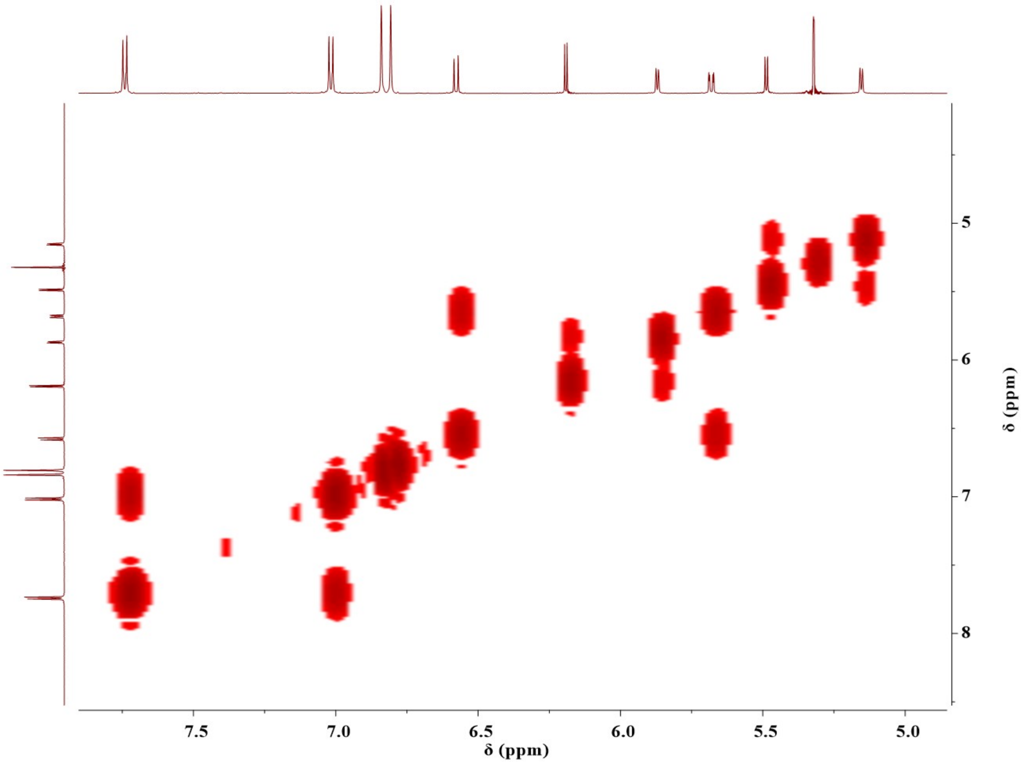

Supplementary Figure 23. Partial 2D-COSY spectrum of compound **5** in CD_2Cl_2.

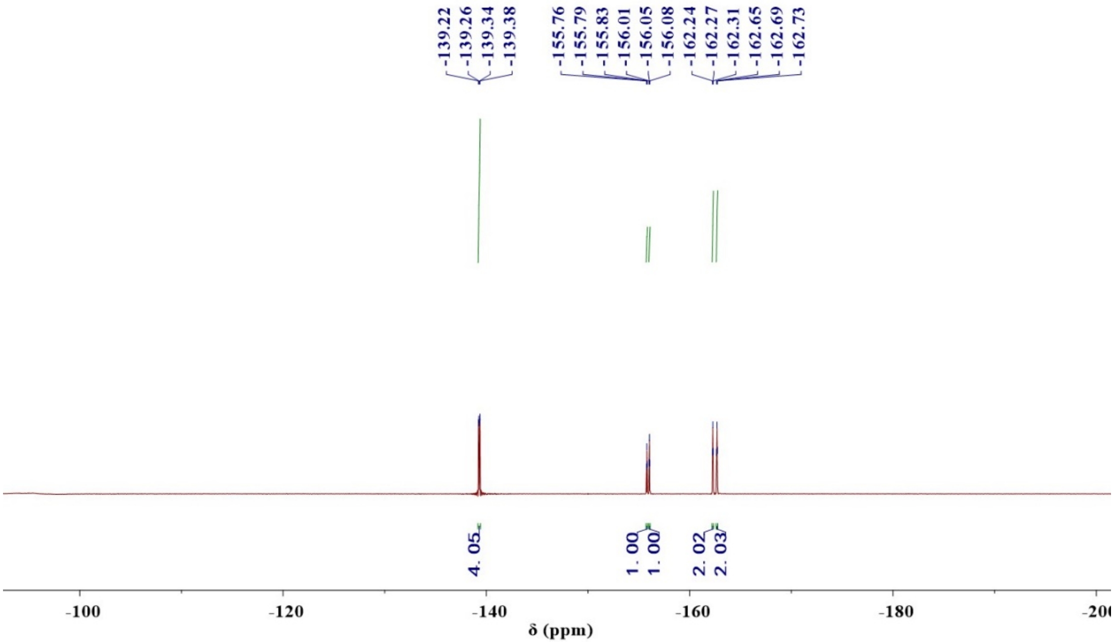

Supplementary Figure 24. ^{19}F NMR spectrum of **5** in CD_2Cl_2.

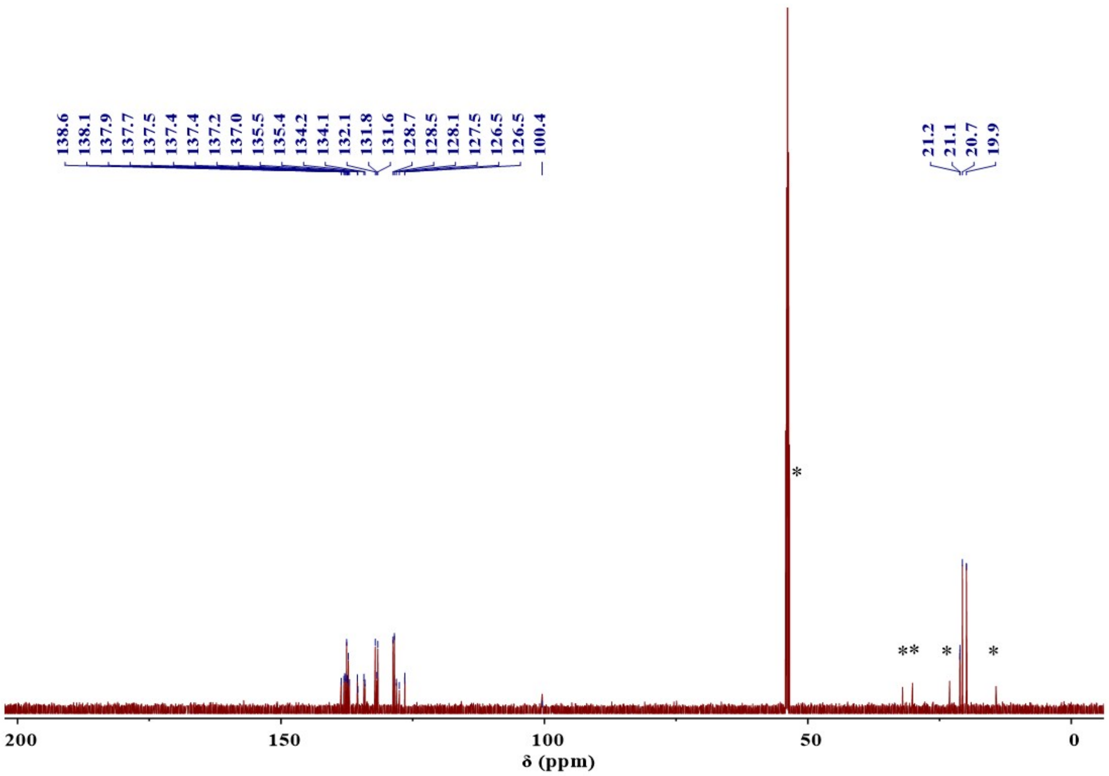

Supplementary Figure 25. ^{13}C NMR spectrum of **5** in CD_2Cl_2. Asterisks indicate residual peaks arising from the solvent or impurities.

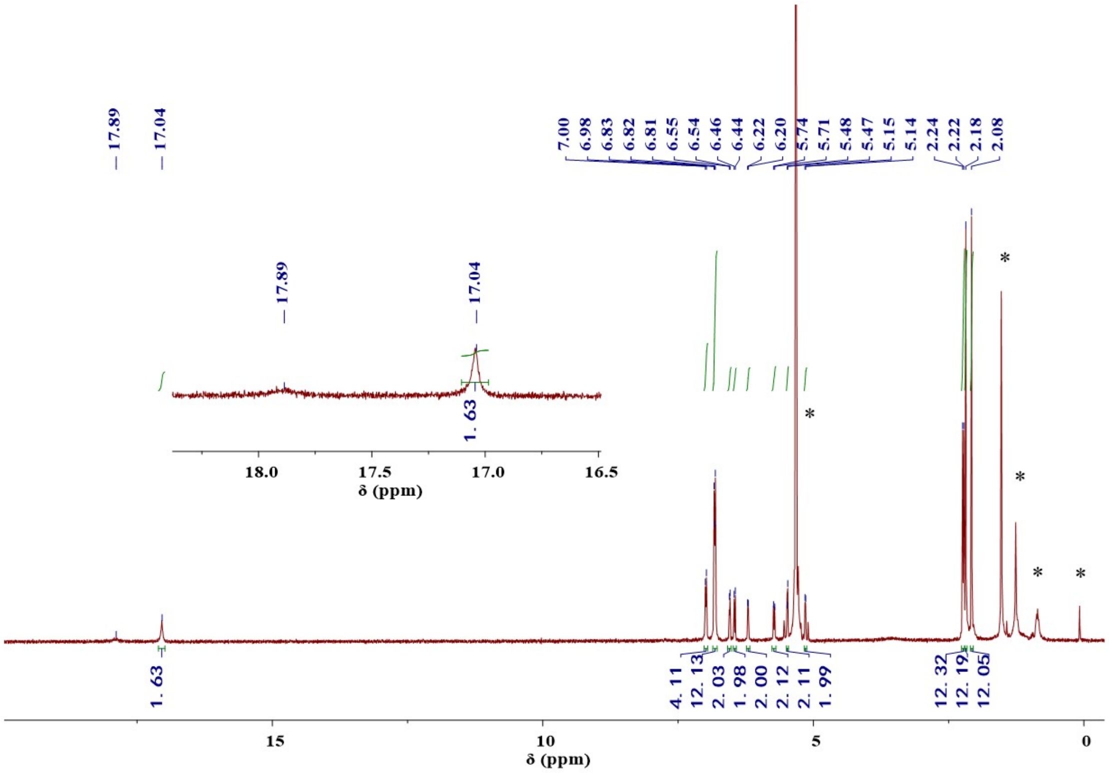

Supplementary Figure 26. ^1H NMR spectrum of **6** in CD_2Cl_2. Asterisks indicate residual peaks arising from the solvent or impurities.

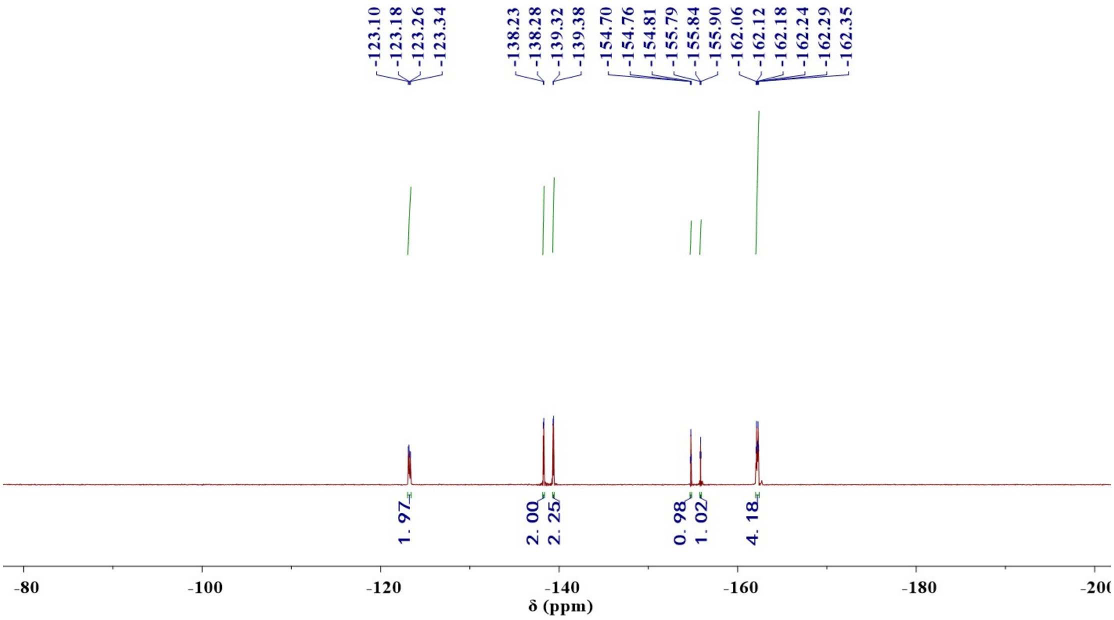

Supplementary Figure 27. ^{19}F NMR spectrum of **6** in CD_2Cl_2.

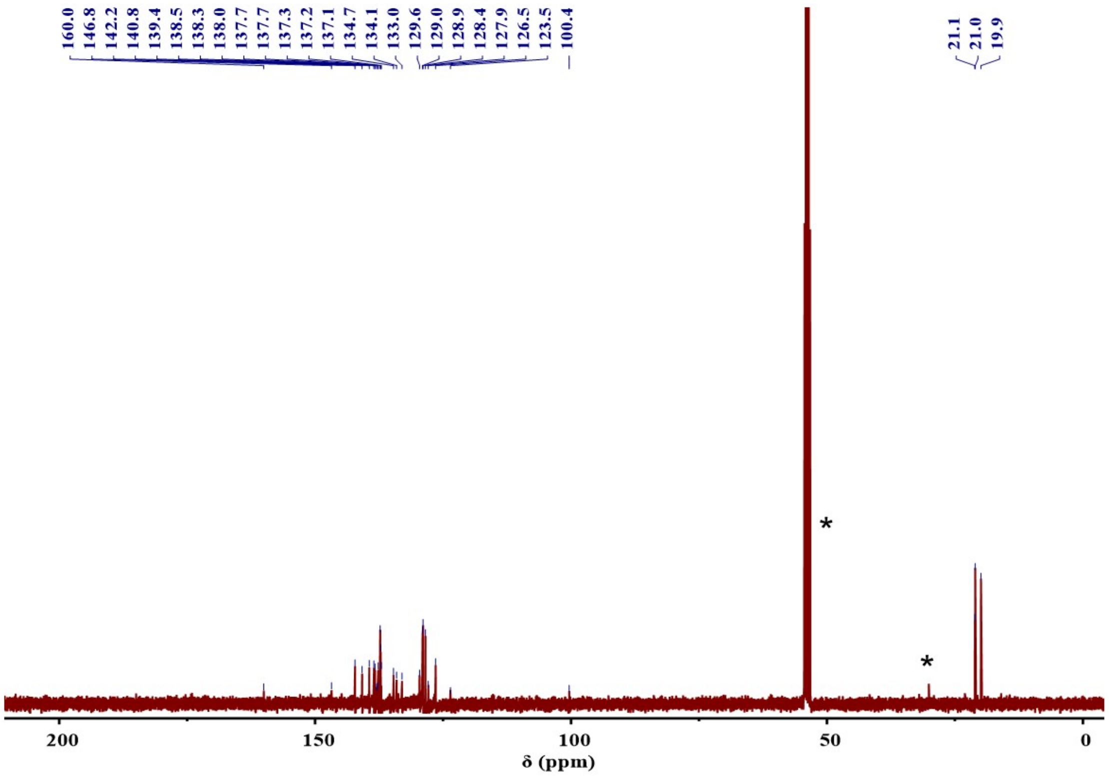

Supplementary Figure 28. ^{13}C NMR spectrum of **6** in CD_2Cl_2. Asterisks indicate residual peaks arising from the solvent or impurities.

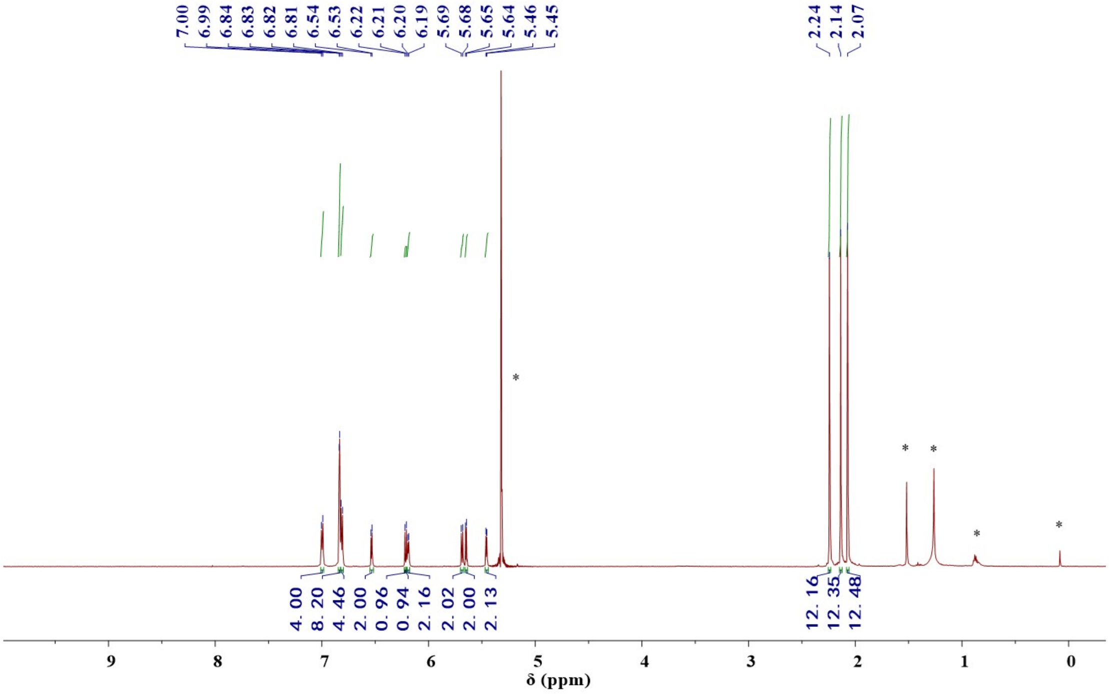

Supplementary Figure 29. ^{1}H NMR spectrum of **7** in CD_2Cl_2. Asterisks indicate residual peaks arising from the solvent or impurities.

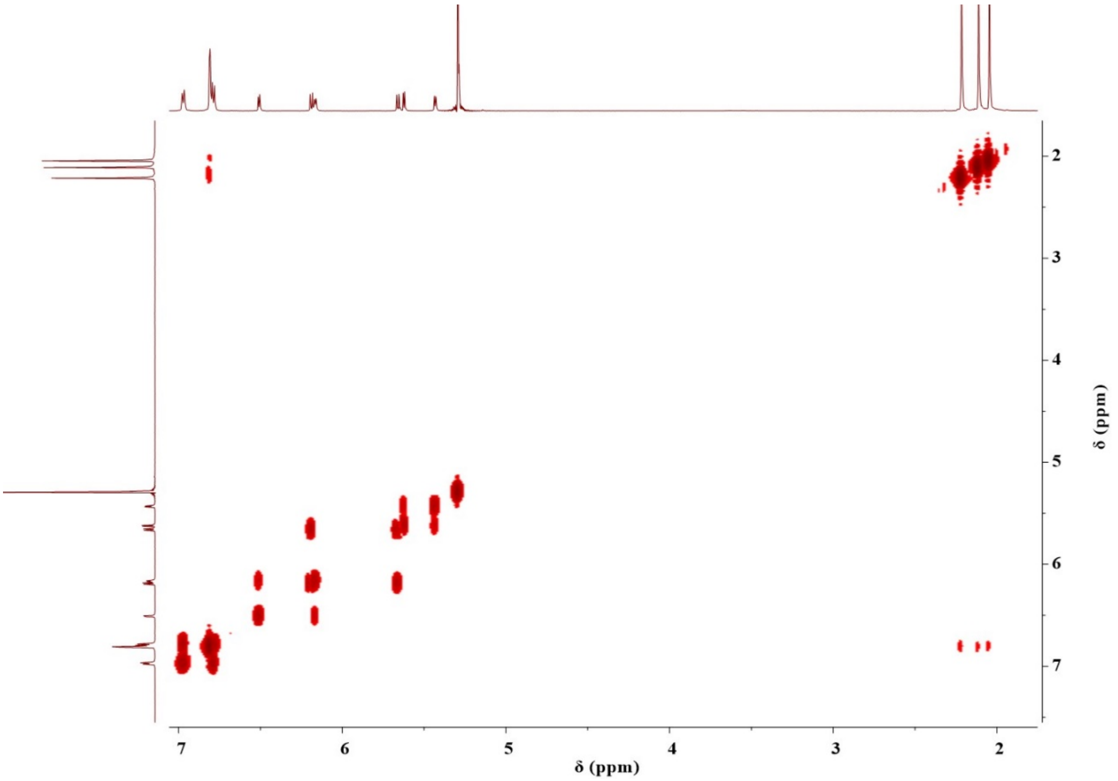

Supplementary Figure 30. 2D-COSY spectrum of **7** in CD_2Cl_2.

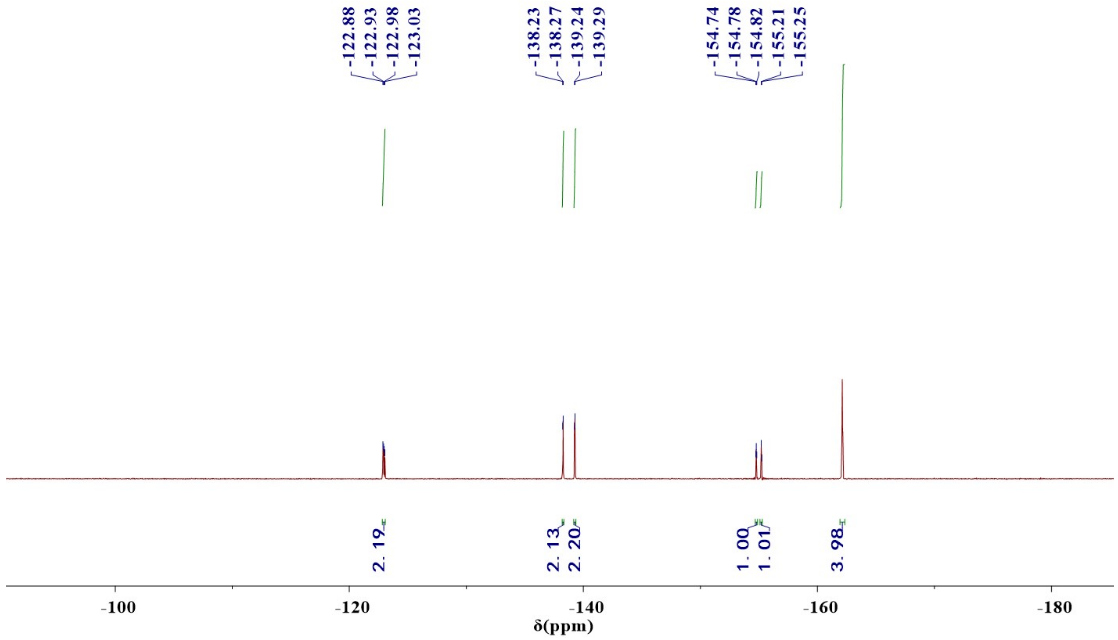

Supplementary Figure 31. ^{19}F NMR spectrum of **7** in CD_2Cl_2.

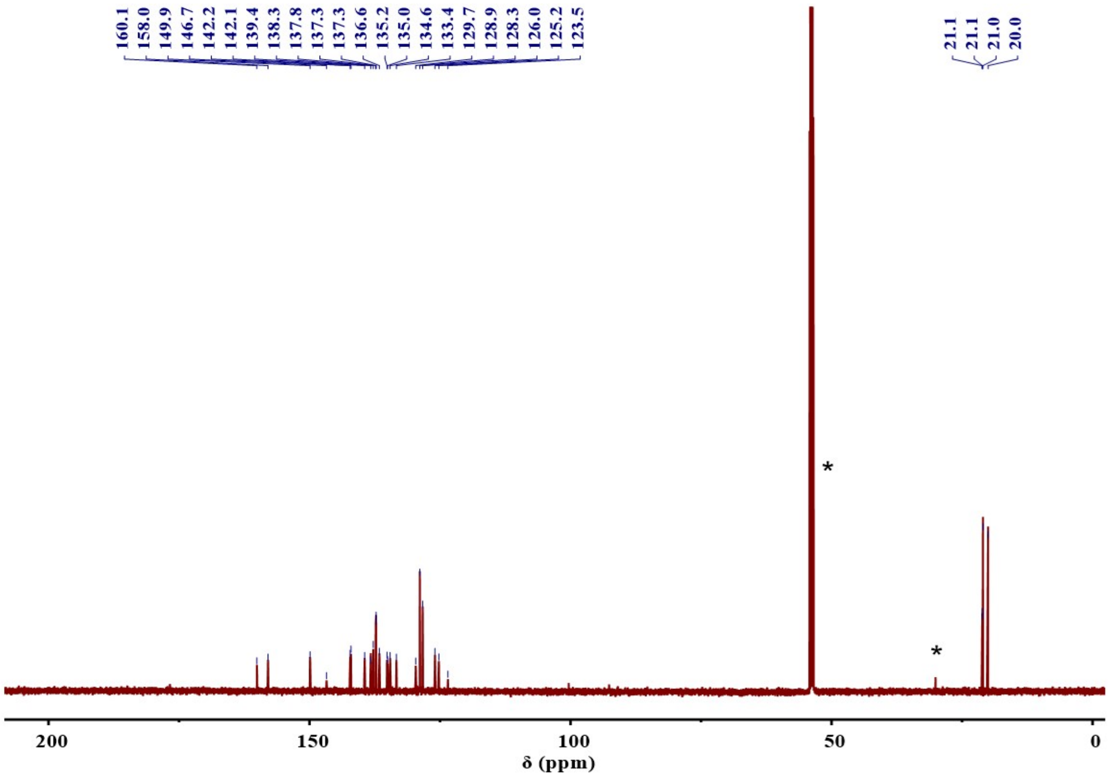

Supplementary Figure 32. ^{13}C NMR spectrum of **7** in CD_2Cl_2. Asterisks indicate residual peaks arising from the solvent or impurities.

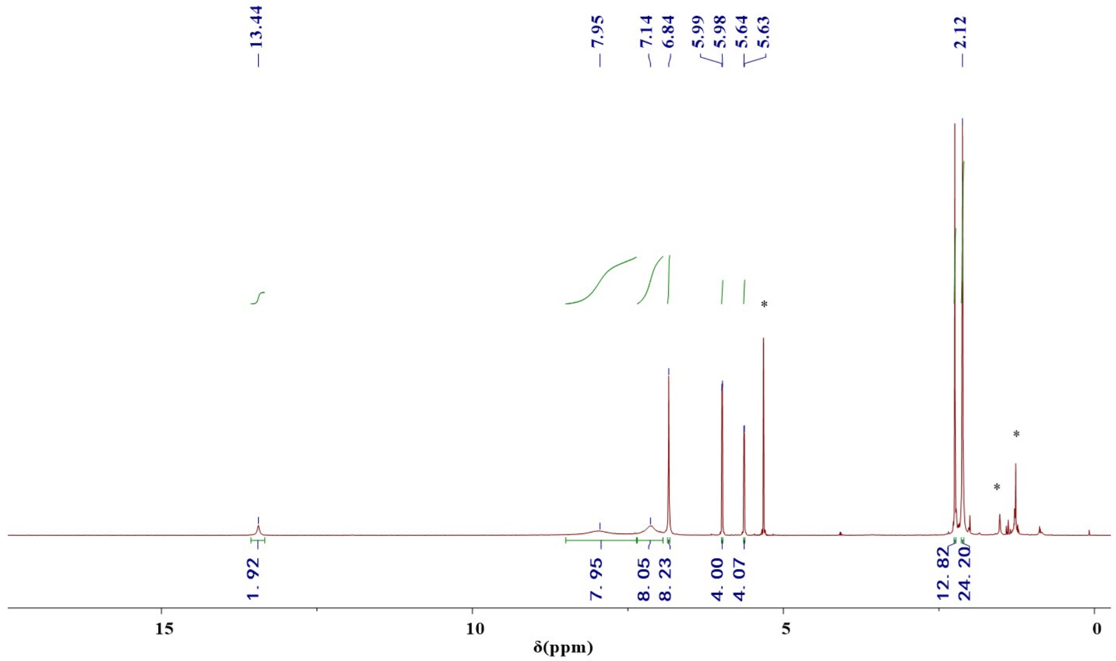

Supplementary Figure 33. ^{1}H NMR spectrum of **8** in CD_2Cl_2. Asterisks indicate residual peaks arising from the solvent or impurities.

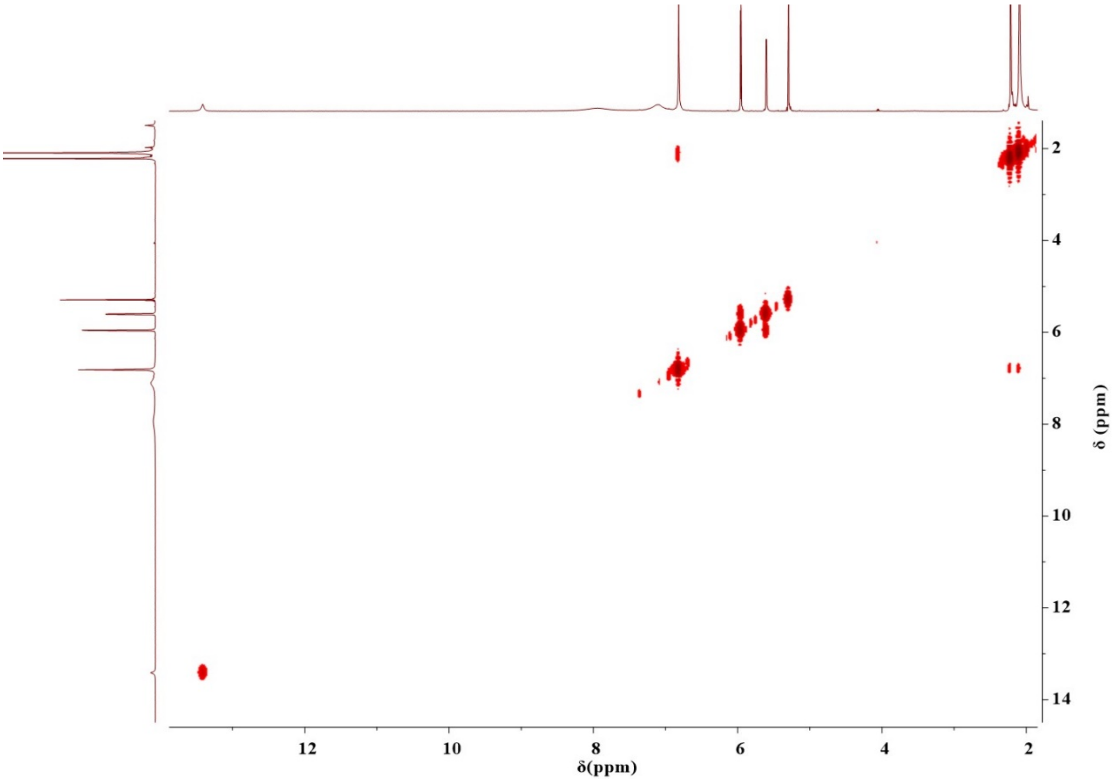

Supplementary Figure 34. 2D-COSY spectrum of **8** in CD_2Cl_2.

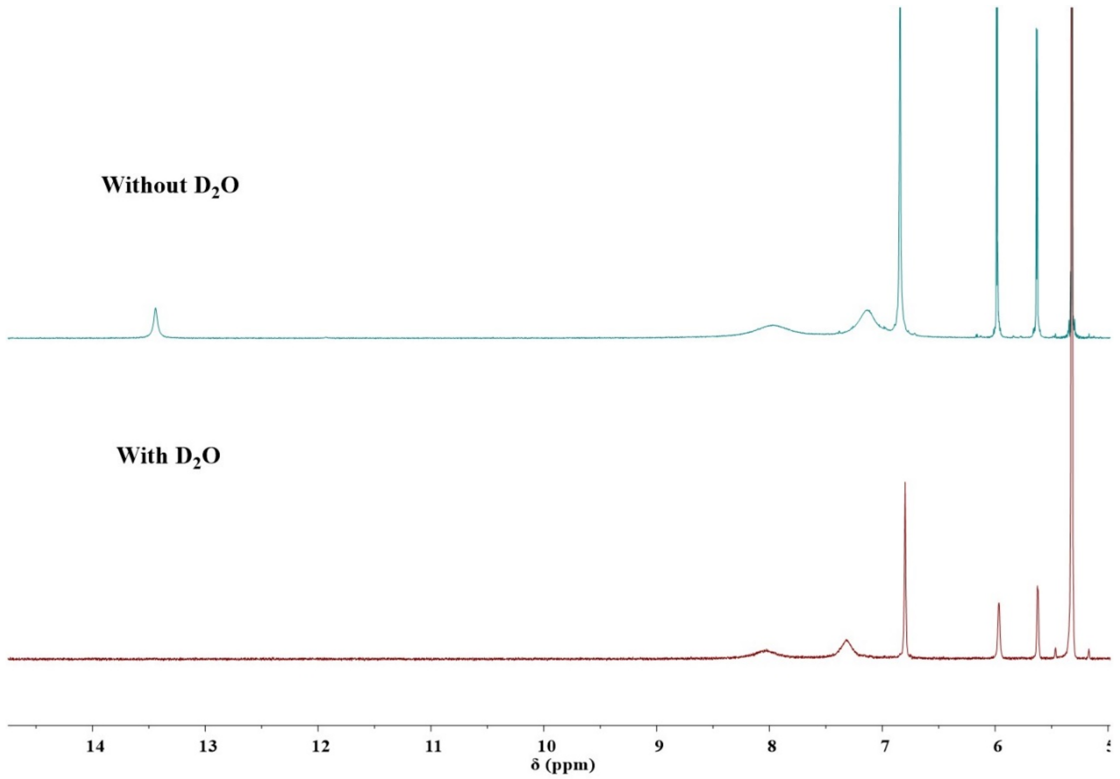

Supplementary Figure 35. Partial ^{1}H NMR spectrum of **8** without and with D_2O in CD_2Cl_2.

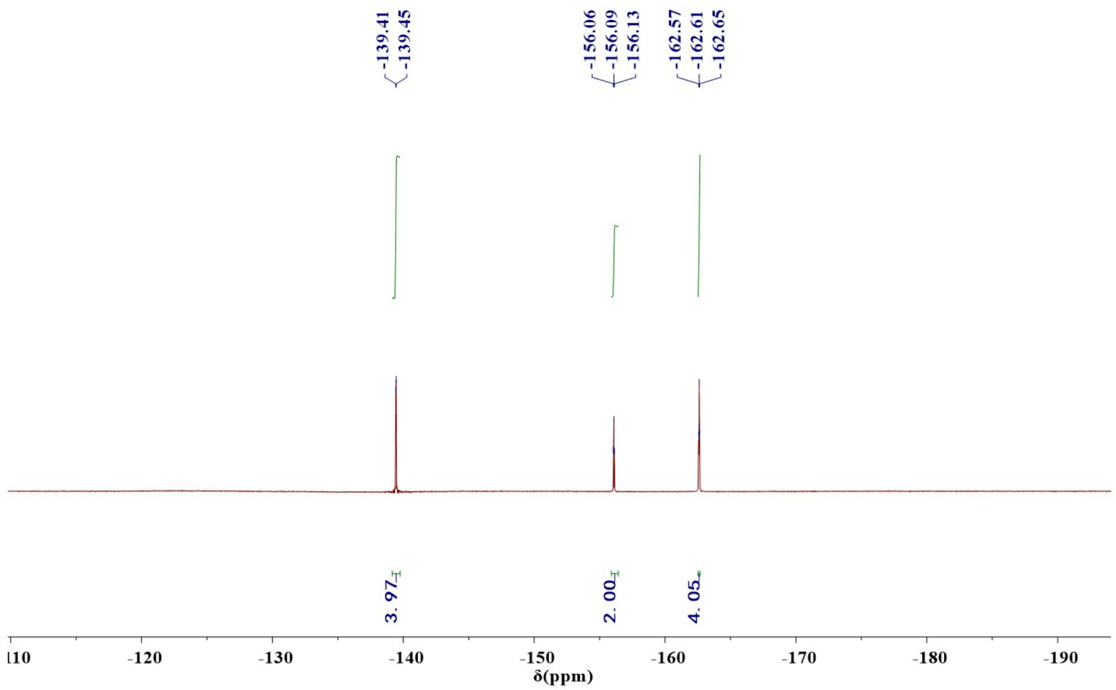

Supplementary Figure 36. ^{19}F NMR spectrum of **8** in CD_2Cl_2.

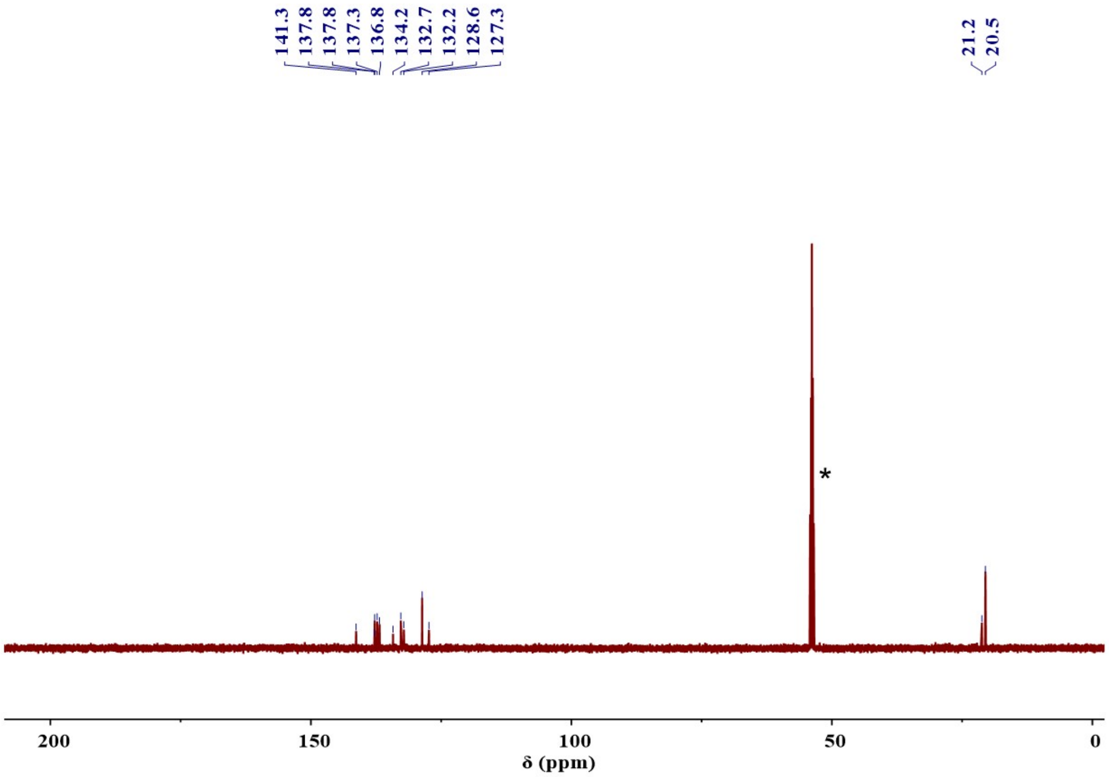

Supplementary Figure 37. ^{13}C NMR spectrum of **8** in CD_2Cl_2. Asterisks indicate residual peaks arising from the solvent or impurities.

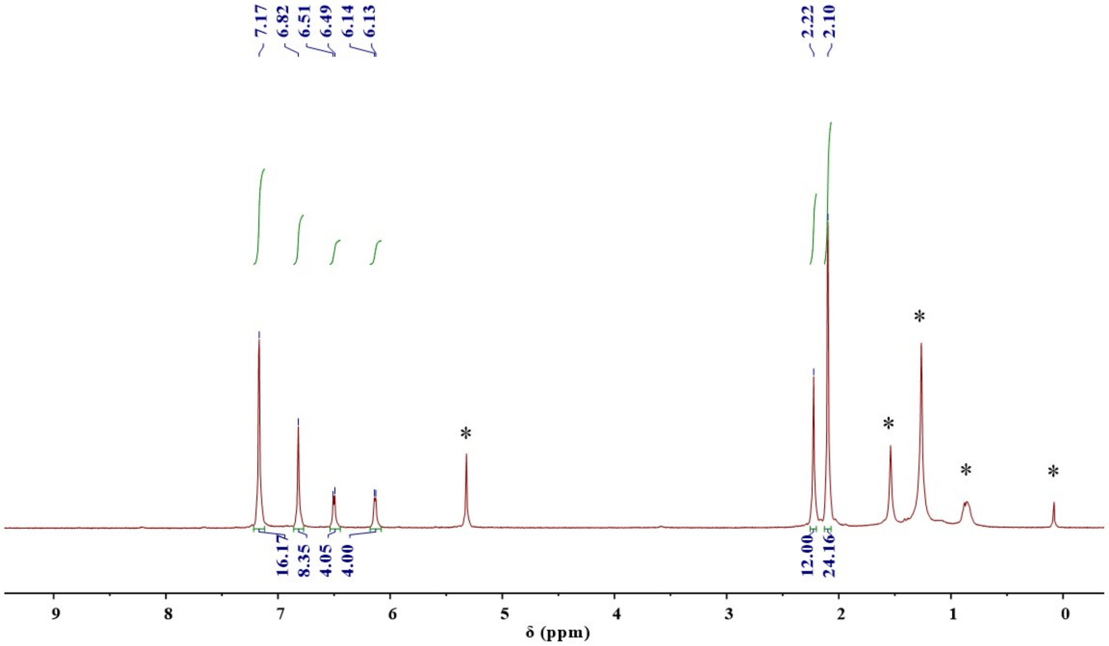

Supplementary Figure 38. ^{1}H NMR spectrum of **10** in CD_2Cl_2. Asterisks indicate residual peaks arising from the solvent or impurities.

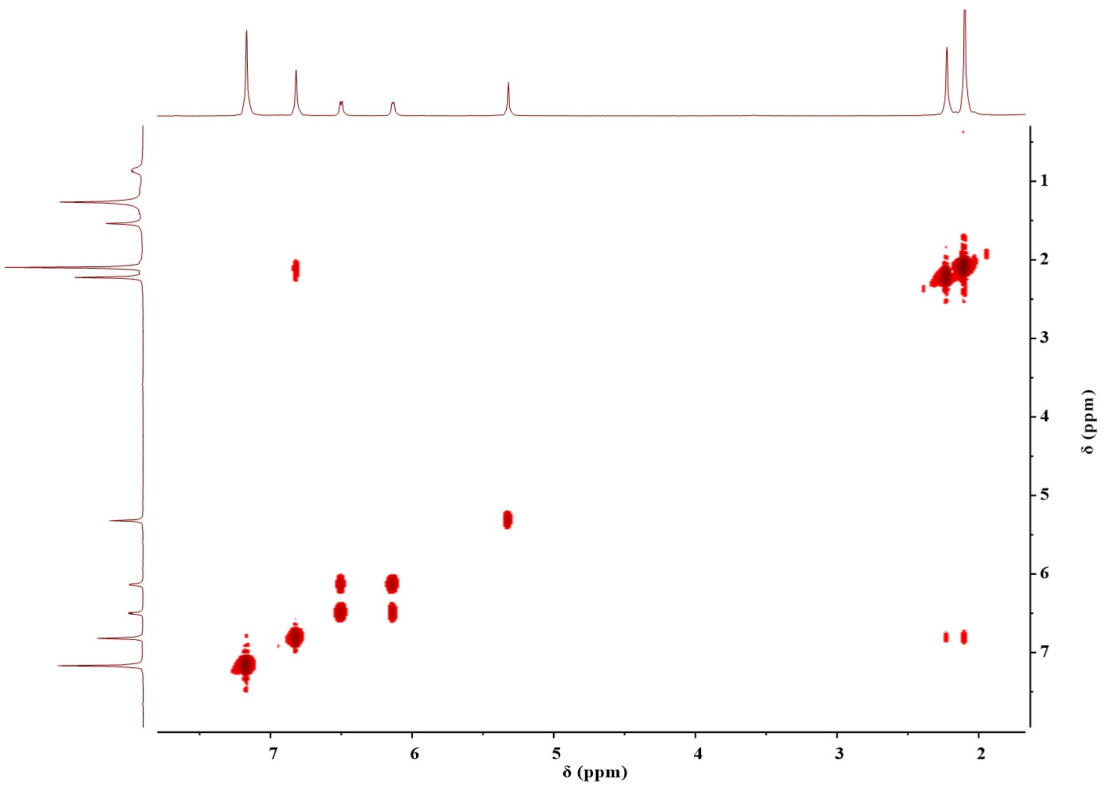

Supplementary Figure 39. 2D-COSY spectrum of **10** in CD_2Cl_2.

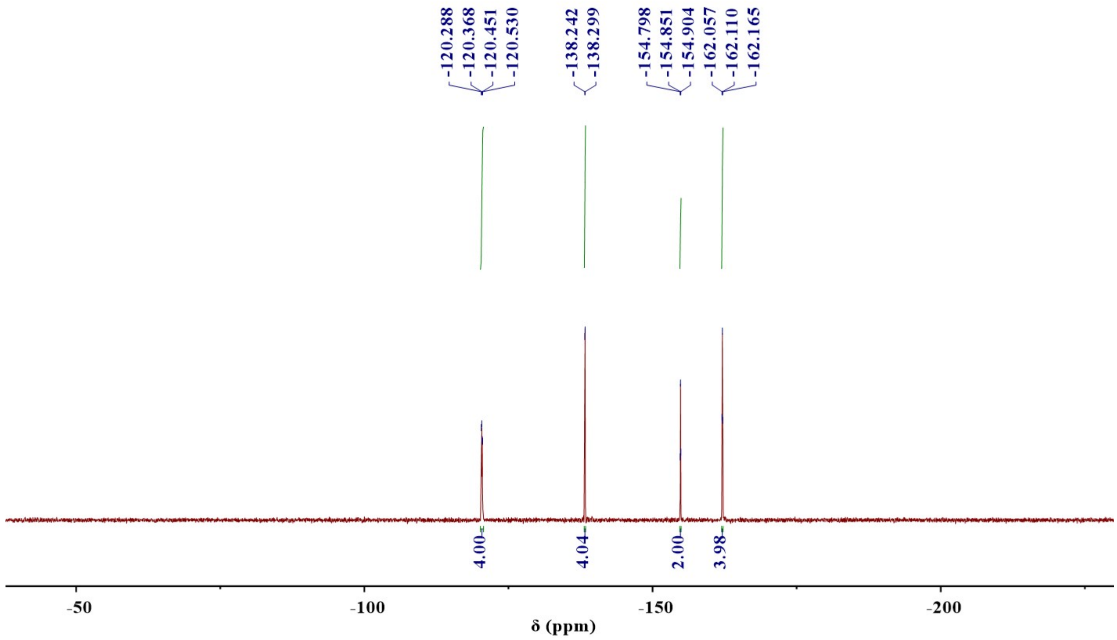

Supplementary Figure 40. ^{19}F NMR spectrum of **10** in CD_2Cl_2.

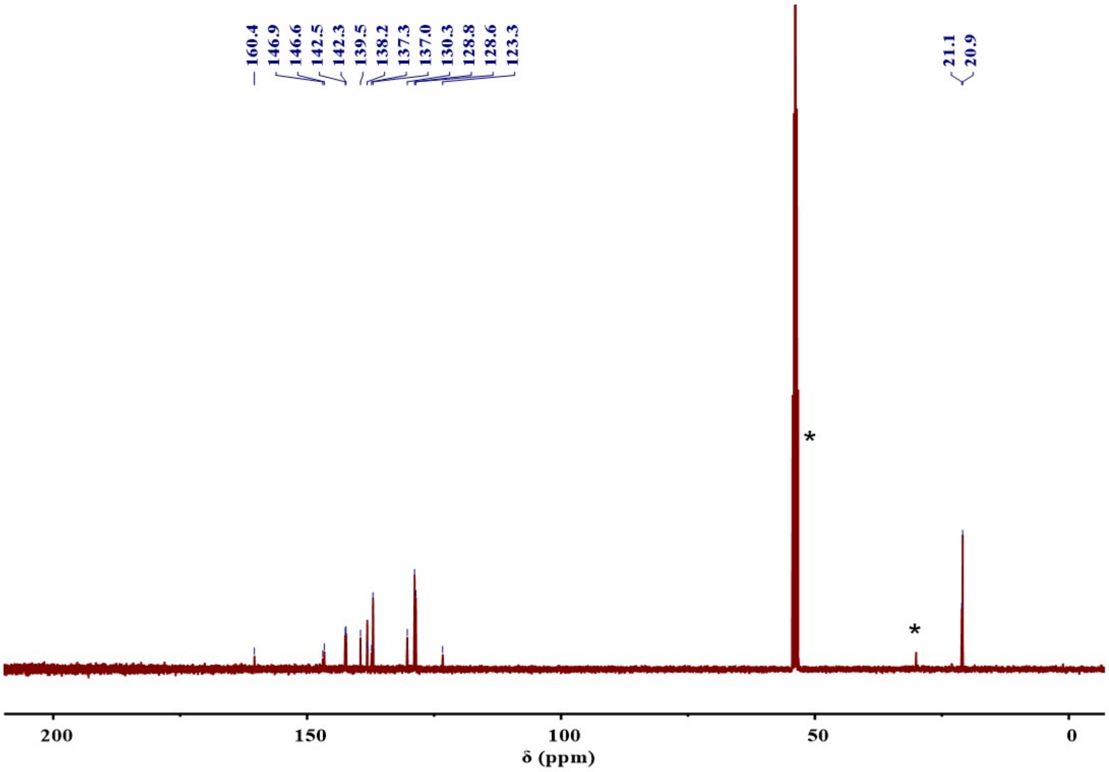

Supplementary Figure 41. ^{13}C NMR spectrum of **10** in CD_2Cl_2. Asterisks indicate residual peaks arising from the solvent or impurities.

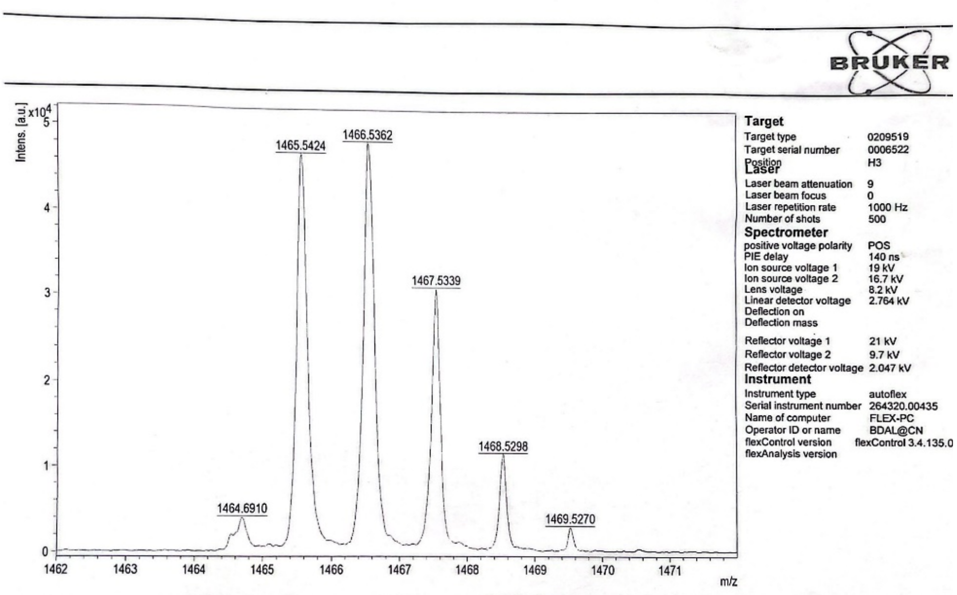

Supplementary Figure 42. HR-MALDI-TOF-MS spectrum of **5**.

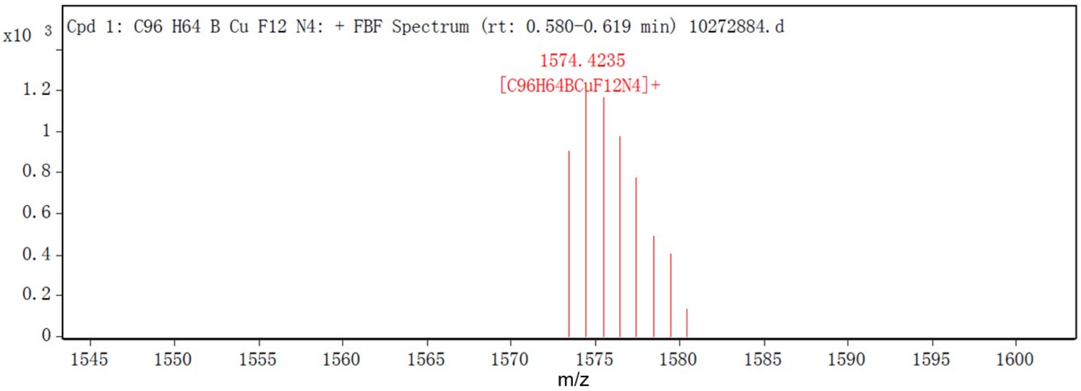

Supplementary Figure 43. HR-ESI-MS spectrum of **7**.

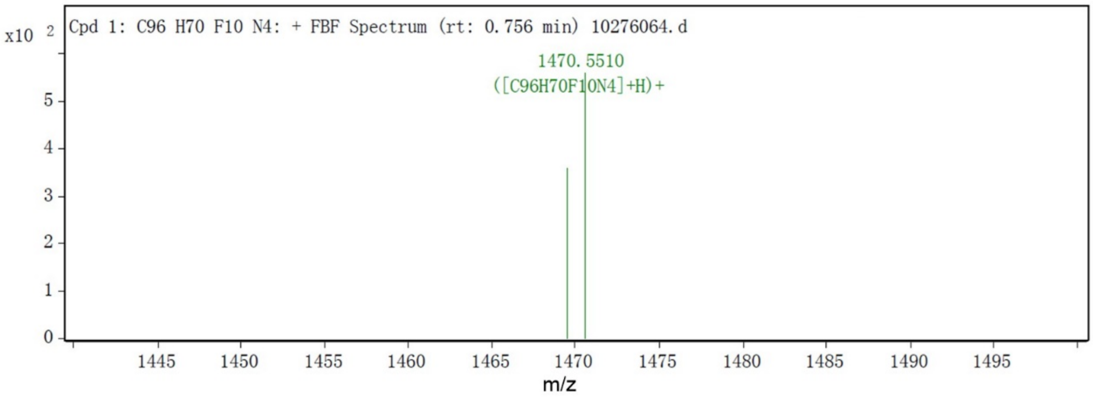

Supplementary Figure 44. HR-ESI-MS spectrum of **8**.

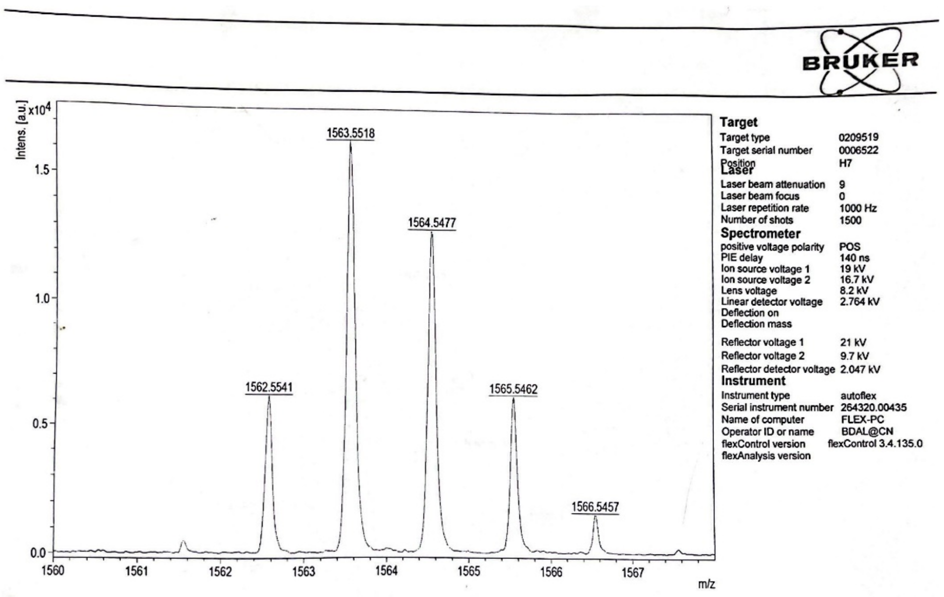

Supplementary Figure 45. HR-MALDI-TOF-MS spectrum of **10**.

7. Supplementary references

1. Gaussian 16, Revision C.01, Frisch, M. J.; Trucks, G. W.; Schlegel, H. B.; Scuseria, G. E.; Robb, M. A.; Cheeseman, J. R.; Scalmani, G.; Barone, V.; Petersson, G. A.; Nakatsuji, H.; Li, X.; Caricato, M.; Marenich, A. V.; Bloino, J.; Janesko, B. G.; Gomperts, R.; Mennucci, B.; Hratchian, H. P.; Ortiz, J. V.; Izmaylov, A. F.; Sonnenberg, J. L.; Williams-Young, D.; Ding, F.; Lipparini, F.; Egidi, F.; Goings, J.; Peng, B.; Petrone, A.; Henderson, T.; Ranasinghe, D.; Zakrzewski, V. G.; Gao, J.; Rega, N.; Zheng, G.; Liang, W.; Hada, M.; Ehara, M.; Toyota, K.; Fukuda, R.; Hasegawa, J.; Ishida, M.; Nakajima, T.; Honda, Y.; Kitao, O.; Nakai, H.; Vreven, T.; Throssell, K.; Montgomery, J. A., Jr.; Peralta, J. E.; Ogliaro, F.; Bearpark, M. J.; Heyd, J. J.; Brothers, E. N.; Kudin, K. N.; Staroverov, V. N.; Keith, T. A.; Kobayashi, R.; Normand, J.; Raghavachari, K.; Rendell, A. P.; Burant, J. C.; Iyengar, S. S.; Tomasi, J.; Cossi, M.; Millam, J. M.; Klene, M.; Adamo, C.; Cammi, R.; Ochterski, J. W.; Martin, R. L.; Morokuma, K.; Farkas, O.; Foresman, J. B.; Fox, D. J. 2016. Gaussian, Inc., Wallingford CT.
2. Chai, J.-D. & Head-Gordon, M. Long-range corrected hybrid density functionals with damped atom–atom dispersion corrections. *Phys. Chem. Chem. Phys.* **10**, 6615-6620, (2008).
